# Novel piperine-carboximidamide hybrids: design, synthesis, and antiproliferative activity via a multi-targeted inhibitory pathway

**DOI:** 10.1080/14756366.2022.2151593

**Published:** 2022-11-30

**Authors:** Lamya H. Al-Wahaibi, Mohamed A. Mahmoud, Yaser A. Mostafa, Ali E. Raslan, Bahaa G. M. Youssif

**Affiliations:** a Department of Chemistry, College of Sciences, Princess Nourah bint Abdulrahman University, Riyadh, Saudi Arabia; b Pharmaceutical Organic Chemistry Department, Faculty of Pharmacy, Assiut University, Assiut, Egypt; c Department of Pharmacognosy, Faculty of Pharmacy, Al-Azhar University, Assiut, Egypt

**Keywords:** Piperine, amidoxime, hybridisation, antiproliferative, kinases

## Abstract

A new series of piperine-carboximidamide hybrids VIa-k was developed as a new cytotoxic agent targeting EGFR, BRAF, and CDK2. The antiproliferative effect against four cancer cells was investigated against erlotinib. Hybrids **VIc**, **VIf**, **VIg**, **VIi**, and **VIk** have the highest antiproliferative activity. Compounds **VIc**, **VIf**, **VIg**, **VIi**, and **VIk** inhibited EGFR with IC_50_ values ranging from 96 to 127 nM. Compounds **VIf** and **VIk** had the most potent inhibitory activity as BRAF^V600E^ (IC_50_ = 49 and 40 nM, respectively) and were discovered to be potent inhibitors of cancer cell proliferation (GI_50_ = 44 and 35 nM against four cancer cell lines, respectively). Compound **VIk**, the most effective derivative as an antiproliferative agent, demonstrated potent anti-CDK2 action with an IC_50_ value of 12 nM, which is 1.5-fold more potent than the reference dinaciclib. Finally, **VIc**, **VIf**, and **VIk** have a high capacity to inhibit LOX-IMVI cell line survival.

## Graphical Abstract

**Figure:**
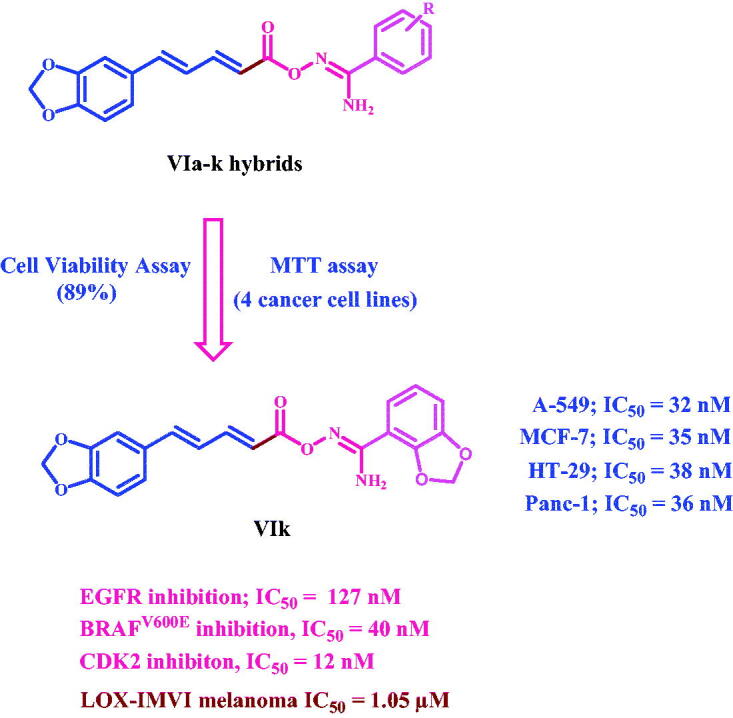

**HIGHLIGHTS**
- A series of piperine-carboximidamide hybrids **VIa-k** was designed and synthesised.
- The new compounds were evaluated as antiproliferative agent targeting EGFR, BRAF, and CDK2.
- Antiproliferative activities were evaluated against four human cancer cell lines.
- Molecular docking studies were carried out against EGFR, mutated BRAF and CDK2-TK.
- *In silico* ADME/pharmacokinetic analysis was considered.

## Introduction

Because cancer is one of the world’s top causes of death, identifying and developing new effective anticancer drugs is one of the most difficult concerns in drug development^1–5^.

A single-targeted treatment method that results in chemotherapy resistance has recently been extensively described^6^. This problem is addressed by a combination treatment that has received clinical approval^7^. Despite the fact that combination therapy offers the potential for extra and potentially synergistic benefits, it typically results in unanticipated side effects such as increased toxicity. Dual or multi-target medications with lower drug interaction risk, improved pharmacokinetics (PK), and safety profiles might be utilised as an alternative to combination therapy. A dual or multiple target kinases can also help to minimise poor patient adherence, off-target effects, medication interactions, and excessive manufacturing costs^8^.

Natural products are a major constant source of lead medicines. Natural product-based developments, including anticancer medicines such as docetaxel, topotecan, and etoposide have been reported^9^^,^^10^. Natural products derived from bacteria, fungi, marine organisms, plants, and animals, as well as natural product-inspired compounds, have shown promising results in clinical studies, including anticancer treatments^11^. Natural product-derived derivatives are thought to account for more than half of anticancer medicines; around 74% of anticancer drugs are either natural or natural product-inspired compounds^12^.

**Figure 1.**
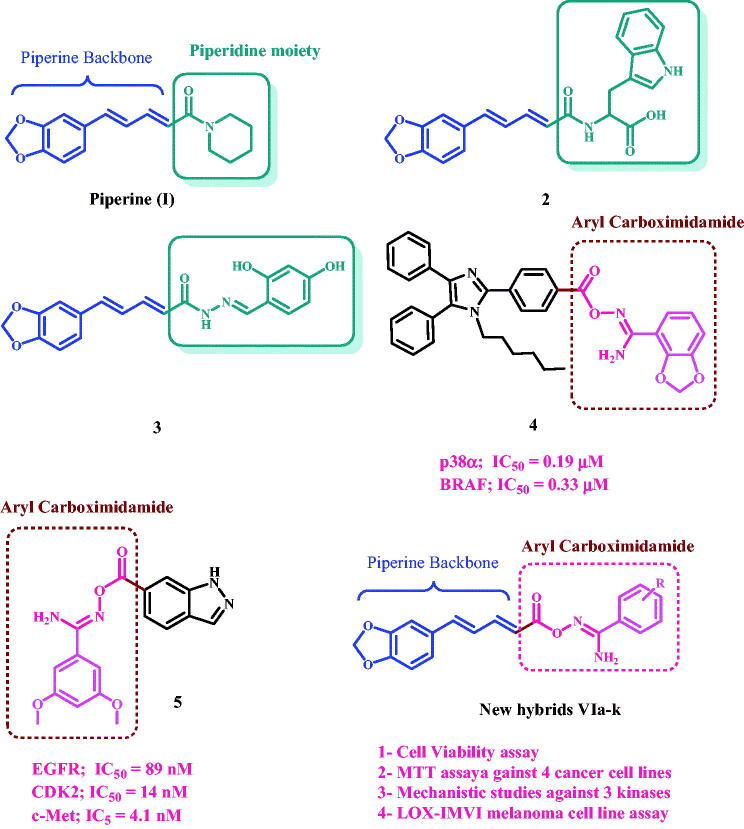
Structures of piperine, compounds **2–5**, and newly designed compounds **VIa-k**.

Piperine **I**, Figure 1, is found in black pepper powder fruit and is widely utilised as a food flavour in a variety of cultures, as well as in many traditional food preservation systems and traditional treatments^13^. Piperine has been shown to have anticancer effect through a variety of pathways. The substitution of the piperidine moiety with other pharmacophoric groups has been shown to increase activity and potency, as demonstrated by compounds **2** and **3**^9^^,^^14^.

Amidoxime-containing compounds, on the other hand, demonstrated strong pharmacological effects, with anticancer activity topping the list^15–18^. The amidoxime moiety has been included as carboxylic and ester group bioisosteres, with the goal of developing prodrugs with enhanced pharmacokinetic and pharmacodynamic properties^19^. Furthermore, amidoxime moiety has demonstrated its capacity to release NO *in vivo* for the purposes of developing nitric oxide-releasing prodrugs^20^.

We recently reported on the design, synthesis, and *in vitro* assessment of a novel series of aryl carboximidamides as dual p38α/BRAF^V600E^ inhibitors. The results revealed that the presence of the carboximidamide moiety is essential for activity, and the best activity correlates with compound **4**, Figure 1. Compound **4** showed significant antiproliferative action against a panel of cancer cell lines. It has an IC_50_ of 0.33 µM and 0.19 µM against BRAF^V600E^ and p38α, respectively^21^.

Another novel series in which the indazole moiety is substituted at the 6-position with aryl and or/heteroaryl carboximidamide moiety was developed in our lab^22^. Compound **5** (Figure 1) was the most active synthetic derivative, with a GI_50_ value of 0.77 µM against the tested four cancer cell lines, compared to the control doxorubicin (GI_50_ = 1.10 µM). Compound **5** inhibited EGFR, CDK2, and c-Met with IC_50_ values of 89 nM, 14 nM, and 4.1 nM, respectively. Furthermore, Compound **5** induced apoptosis by increasing cytochrome C levels and activating the intrinsic apoptotic pathway.

Motivated by the antiproliferative effects of piperine (**I**), compounds **2** and **3**, as well as the promising antiproliferative action of aryl carboximidamide derivatives **4** and **5**, we present here the design, synthesis, and antiproliferative action of a new hybrid scaffold **VIa-k** (Figure 1), in which the aryl carboximidamide pharmacophore was bound to the piperine backbone using molecular hybridisation approach in order to obtain potent antiproliferative agents. The new hybrids will be tested against four cancer cell lines using the MTT assay to determine their IC_50_. The most effective hybrids will be tested further for mechanistic action against EGFR, BRAF, and CDK2. Furthermore, the most potent derivatives as BRAF inhibitors will be tested for their inhibitory action against the LOX-IMVI melanoma cell line, which has BRAF^V600E^ kinase overexpression. Finally, docking studies were performed on the most active derivatives against the active sites of EGFR, BRAF, and CDK2.

## Results and discussion

### Chemistry

**Scheme 1:**
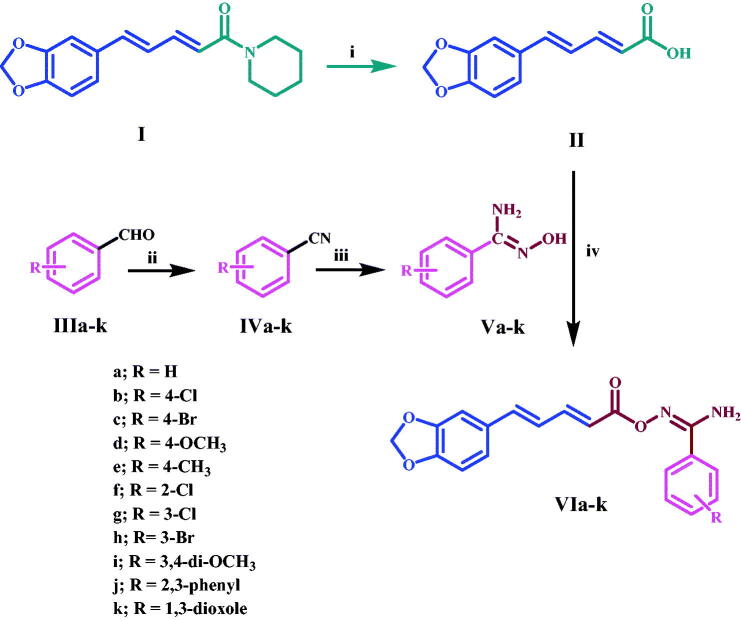
Synthesis of target compounds **VIa-k. Reagents and reaction conditions**: i) KOH, Ethanol, reflux 12 h; ii) NH_3_, THF, I_2_, r.t. 3 h, 65–80%; iii) NH_2_OH, methanol, NaHCO_3_, reflux 3–5 h, 80–90%; iv) CDI, Acetonitrile, r.t. 3 h.

Scheme 1 depicts the synthetic steps for the key intermediates **II**, **Va-k**, and target compounds **VIa-k**. Piperine **I** was extracted, purified, and crystallised from natural sources using the methods described^23^. Piperic acid **II** was synthesised by hydrolysing piperine **I** with alcoholic KOH^24^, as shown in Scheme 1. Amidoximes **Va-l** were synthesised as described^25^ and then coupled with piperic acid **II** using CDI at room temperature for 3 h to obtain the target compounds **VIa-k**. ^1^H NMR, ^13^C NMR, and elemental microanalyses were used to confirm all of the aryl carboximidamides **VIa-k**. The IR spectrum of **VId**, a prominent example of this series, shows the presence of peaks at 3498 and 3381 (NH_2_), 1760 (C = O), and 1622 (C = N). The ^1^H NMR spectrum of this compound revealed the appearance of new two doublet signals with *J* = 8.5, 8.7 Hz, corresponding to para disubstituted pattern of amidoxime moiety at δ 7.69 and δ 7 ppm, respectively, as well as the appearance of new broad singlet NH_2_ at δ 6.76 ppm and finally the appearance of singlet signal of methoxy (OCH_3_) group at δ 3.8 ppm. The presence of a new carbonyl (C = O) group signal at 161.4 ppm in the ^13^C NMR spectra of this molecule confirmed the earlier observations, as did the appearance of a methoxy (OCH_3_) group signal at 55.7 ppm.

### Biology

#### In vitro anticancer activity

##### Cell viability assay

A human mammary gland epithelial (MCF-10A) normal cell line was used to perform the viability test. The viability of compounds **VIa-k** was determined using the MTT assay after 4 days of incubation with MCF-10A cells^26^^,^^27^. The results showed that none of the compounds tested were cytotoxic, and that majority of the compounds tested at 50 µM had cell viability more than 85%.

##### Antiproliferative activity

Compounds **VIa-k** were investigated for antiproliferative efficacy against four human cancer cell lines using the MTT assay and erlotinib as the reference drug: Panc-1 (pancreatic cancer cell line), MCF-7 (breast cancer cell line), HT-29 (colon cancer cell line), and A-549 (epithelial cancer cell line)^28^^,^^29^. Table 1 shows the median inhibitory concentration (IC_50_) calculated using Graph Pad Prism software (Graph Pad Software, San Diego, CA, USA). The average (GI_50_) versus the four cancer cell lines was chosen for ease of manipulation.

**Table 1.** Antiproliferative activity of compounds **VIa-k** and **Erlotinib**. 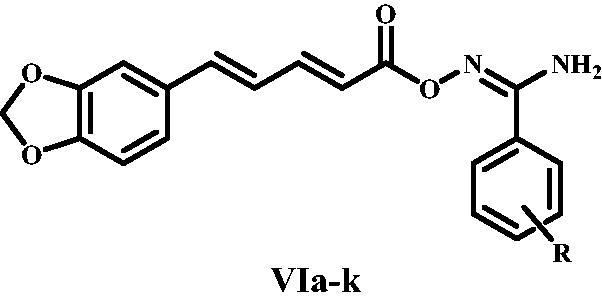

Comp.	Cell viability %	R	Antiproliferative activity IC_50_ ± SEM (nM)				
			A-549	MCF-7	Panc-1	HT-29	Average (GI_50_)
Via	89	H	95 ± 10	97 ± 10	104 ± 10	105 ± 10	100
VIb	85	4-Cl	55 ± 6	57 ± 6	59 ± 6	58 ± 6	57
Vic	86	4-Br	45 ± 5	47 ± 5	53 ± 5	55 ± 5	50
Vid	91	4-OCH_3_	50 ± 5	52 ± 5	57 ± 5	57 ± 5	54
Vie	89	4-CH_3_	69 ± 7	73 ± 7	75 ± 7	75 ± 7	73
VIf	90	2-Cl	41 ± 4	43 ± 4	45 ± 4	47 ± 4	44
VIg	87	3-Cl	42 ± 5	47 ± 5	49 ± 5	49 ± 5	47
VIh	91	3-Br	61 ± 6	64 ± 6	69 ± 6	67 ± 6	65
Vii	89	3,4-diOMe	36 ± 4	39 ± 3	40 ± 4	40 ± 4	39
VIj	85	2,3-phenyl	78 ± 8	81 ± 8	84 ± 8	88 ± 9	83
VIk	89	1,3-dioxole	32 ± 3	35 ± 3	36 ± 3	38 ± 3	35
Erlotinib	–	–	30 ± 3	40 ± 3	30 ± 3	30 ± 3	33

In general, compounds **VIa-k** demonstrated strong antiproliferative activity with GI_50_ values ranging from 35 nM to 100 nM when compared to the reference erlotinib, which had a GI_50_ of 33 nM. The data in Table 1 reveal that **VIa-k** were more effective against epithelial cancer cell line (A-549) than breast cancer cell line (MCF-7).

The 1,3-benzodioxole derivative **VIk** (R = 1,3-dioxole) was the most potent of the synthesised derivatives, with a GI_50_ value of 35 nM, which was comparable to erlotinib and even more potent than erlotinib in MCF-7 cell line with IC_50_ = 35 nM, while for erlotinib IC_50_ = 40 nM.

Compound **VIi** (R = 3,4-di-OMe) ranks second in activity against the cell lines examined, with a GI_50_ of 39 nM. The number of the methoxy groups appears to be important for the antiproliferative action. Compound **VId** (R = 4-OMe), for example, has a GI_50_ of 54 nM, which is about 1.4-fold less potent than the dimethoxy derivative **VIi**. Furthermore, compound **VIj** (R = 2,3-Ph) has a GI_50_ of 83 nM, which is 2-times lower than **VIi**, indicating that the dimethoxy moiety is the most well tolerated.

Compound **VIf** (R = 2-Cl) demonstrated promising antiproliferative activity against the four cancer cell lines, with a GI_50_ of 44 nM. The antiproliferative action appears to be strongly influenced by the position and/or type of halogen atom. The GI_50_ of compounds **VIb** (R = 4-Cl) and **VIg** (R = 3-Cl) was 57 nM and 47 nM, respectively, indicating that when the chlorine atom is the substituent, position 2 on the phenyl group is the optimum for activity. Furthermore, compound **VIc** (R = 4-Br) had a GI_50_ of 50 nM, which is more potent than compound **VIb** (R = 4-Cl), which had a GI_50_ of 57 nM, demonstrating that the bromine atom is more tolerated than the chlorine one in terms of antiproliferative action.

The unsubstituted derivative, compound **VIa** (R = H), was the least potent of all synthesised derivatives, with a GI_50_ of 100 nM, approximately three times less active as erlotinib, highlighting the relevance of phenyl moiety substitution for antiproliferative action.

#### EGFR inhibitory activity

The EGFR-TK test^30^ was used to evaluate the inhibitory potency of the most potent derivatives **VIc**, **VIf**, **VIg**, **VIi**, and **VIk** against EGFR, and the outcomes are displayed in Table 2. Compounds **VIc**, **VIf**, **VIg**, **VIi**, and **VIk** inhibited EGFR with IC_50_ values ranging from 96 to 127 nM. According to the findings, all the tested derivatives were less potent than erlotinib (IC_50_ = 80 ± 5 nM). The 3-chloro phenyl derivative **VIg** (R = 3-Cl) was the most potent of all synthesised derivatives, with an IC_50_ value of 96 nM, which was 1.2-fold lower than erlotinib. The results of the EGFR inhibitory assay test indicate that the EGFR may be a potential target for the antiproliferative effect of some compounds.

**Table 2.** Effects of compounds **VIc**, **VIf**, **VIg**, **VIi**, **Vik**, **Erlotinib, Vemurafenib**, and **Dinaciclib** on EGFR, BRAF^V600E^, and CDK2.

Compound	EGFR inhibition IC_50_ ± SEM (nM)	BRAF^V600E^ inhibition IC_50_ ± SEM (nM)	CDK2 IC_50_ ± SEM (nM)
VIc	112 ± 8	57 ± 6	23 ± 2
VIf	105 ± 7	49 ± 5	21 ± 2
VIg	96 ± 6	63 ± 6	20 ± 2
Vii	120 ± 10	72 ± 7	17 ± 2
VIk	127 ± 10	40 ± 4	12 ± 2
Erlotinib	80 ± 5	60 ± 5	ND
Vemurafenib	ND	30 ± 3	ND
Dinaciclib	ND	ND	20 ± 2

ND: Not Determined

#### BRAF^v600e^ assay

An *in vitro* study was conducted to evaluate the anti-BRAF^V600E^ of **VIc**, **VIf**, **VIg**, **VIi**, and **VIk**^31^. The enzyme assay revealed that the tested five compounds strongly inhibited BRAF^V600E^, with IC_50_ values ranging from 40 to 72 nM (See Table 2). In all cases, the IC_50_ of the investigated compounds are higher than that of vemurafenib (IC_50_ = 30 ± 3). Compounds **VIf** and **VIk** had the most potent inhibitory activity as BRAF^V600E^ (IC_50_ = 49 and 40 nM, respectively) and were discovered to be potent inhibitors of cancer cell proliferation (GI_50_ = 44 and 35 nM, respectively). According to the study’s findings, the examined compounds have substantial antiproliferative activity and are effective at inhibiting BRAF^V600E^.

#### Cdk2 inhibitory assay

Compounds **VIc**, **VIf**, **VIg**, **VIi**, and **VIk** were further investigated for their potential to inhibit the CDK2 enzyme^32^. The IC_50_ values are shown in Table 2. According to the results, all compounds inhibited CDK2 with IC_50_ values ranging from 12 nM to 23 nM in comparison to the reference dinaciclib (IC_50_ = 20 nM). Compound **VIk**, the most effective derivative as an antiproliferative agent, demonstrated potent anti-CDK2 action with an IC_50_ value of 12 nM, which is 1.5-fold more potent than the reference dinaciclib. On the other hand, compounds **VIf**, **VIg**, and **VIi** exhibited significant activity against CDK2 (IC_50_ = 21, 20, and 17 nM) being equipotent to dinaciclib. The results of this assay show that the examined compounds have substantial antiproliferative activity and are effective at inhibiting both CDK2 and BRAF^V600E^.

#### Lox-IMVI melanoma cell line cytotoxicity assay

The MTT cytotoxicity assay was used to assess the anticancer activity of **VIc**, **VIf**, and **VIk** on the LOX-IMVI melanoma cell line, which comprises BRAF^V600E^ kinase overexpression^33^. The compounds were evaluated at 5-dose concentrations to ascertain their IC_50_ values, with staurosporine acting as a control. Table 3 reveals that the derivatives **VIc**, **VIf**, and **VIk** have a high capacity to inhibit LOX-IMVI cell line survival, with IC_50_ values ranging from 1.05 µM to 1.40 µM. In all cases, the tested compounds were at least 5 times more effective than the reference Staurosporine, which had an IC_50_ of 7.10 µM. Compounds **VIf** and **VIf** demonstrated promising cytotoxic activity against LOX-IMVI melanoma cell line with IC_50_ values of 1.10 and 1.05 μM, respectively.

**Table 3.** IC_50_ cytotoxicity of compounds **VIc**, **VIf**, and **VIk** against LOXIMVI melanoma cell line.

Compound	LOX-IMVI melanoma IC_50_ ± SEM (µM)
Vic	1.40 ± 0.02
VIf	1.10 ± 0.01
VIk	1.05 ± 0.01
Staurosporine	7.10 ± 0.05

### Molecular docking simulations

To determine the binding affinity and mode of inhibition of the most active derivatives against potential cellular targets of this class of compounds, we ran in-silico molecular docking simulations for compounds **VIc**, **VIf**, **VIg**, **VIi**, and **VIk** against EGFR, mutated BRAF, and CDK2 proteins. The results were extremely promising, particularly with compounds **VIg**, **VIi**, and **VIk** against all the cellular targets used in this study.

**Figure 2.**
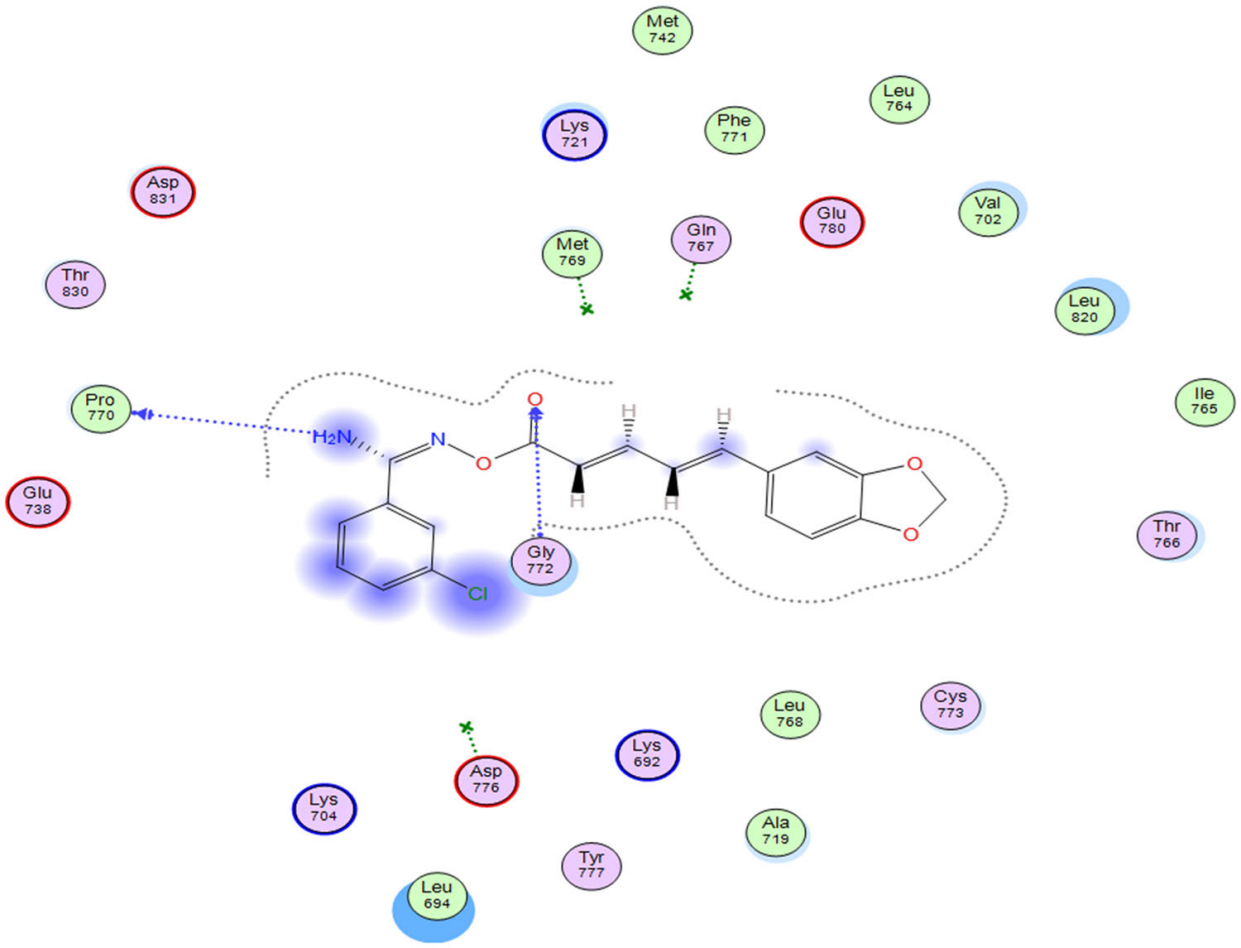
Schematic representation of docking interactions of compound **VIg** within EGFR (PDB ID: 1M17) showing two H-bond interactions with PRO 770 and GLY772 as blue-coloured arrows.

Starting with the RSCB deposited crystal structure of the EGFR protein having Erlotinib as a co-crystallised ligand (PDB ID: 1M17), simulations revealed good docking score (S = −5.84 to −6.72 kcal/mol) with all test compounds as listed in Table S1 (supporting information). Visual inspections of each test molecule’s docking poses revealed numerous interactions with various amino acid residues lining the EGFR active site (Figure 2 and Figure S1). Using compound **VIg** as a model compound (highest docking score; S = −6.72 kcal/mol) to examine its docking poses and gain an understanding of the mode of inhibition of this class of compounds within the EGFR protein, we observed H-bond interactions with PRO770 and GLY772 amino acid residues, as shown in Figure 2.

Although the docking scores of the test molecules were very promising, their binding interactions with various amino acid residues lining the EGFR active site remained random, and none of them managed to show a binding interaction with key amino acids (GLN767 and MET769) as did co-crystallised ligand. All these findings demonstrated that the EGFR protein is not the best active site to explain the mode of inhibition of such compounds as potential anti-proliferative agents.

Docking into BRAF and CDK2 active sites, on the other hand, revealed a significant common bonding interaction between test molecules and the amino acid residues lining these two active sites, as well as at least one or more bonding interactions with key amino acid residues.

Additionally, MTT assay results on the LOX-IMVI melanoma cell line, which is known for being enriched with BRAF^V600E^ kinases, showed promising inhibitory results (5-times better than reference compound; staurosporine). As a result of all these intriguing findings regarding CDK2 and BRAF^V600E^ proteins, we chose to investigate the potential mode of interactions within the active sites of these two proteins.

As shown in Table S1, compounds **VIc**, **VIf**, **VIg**, **VIi**, and **VIk** showed good and comparable docking score and RMSD values to that of co-crystallised ligands. Compound **VIk** showed the best docking score (S) within its congeners

**Figure 3.**
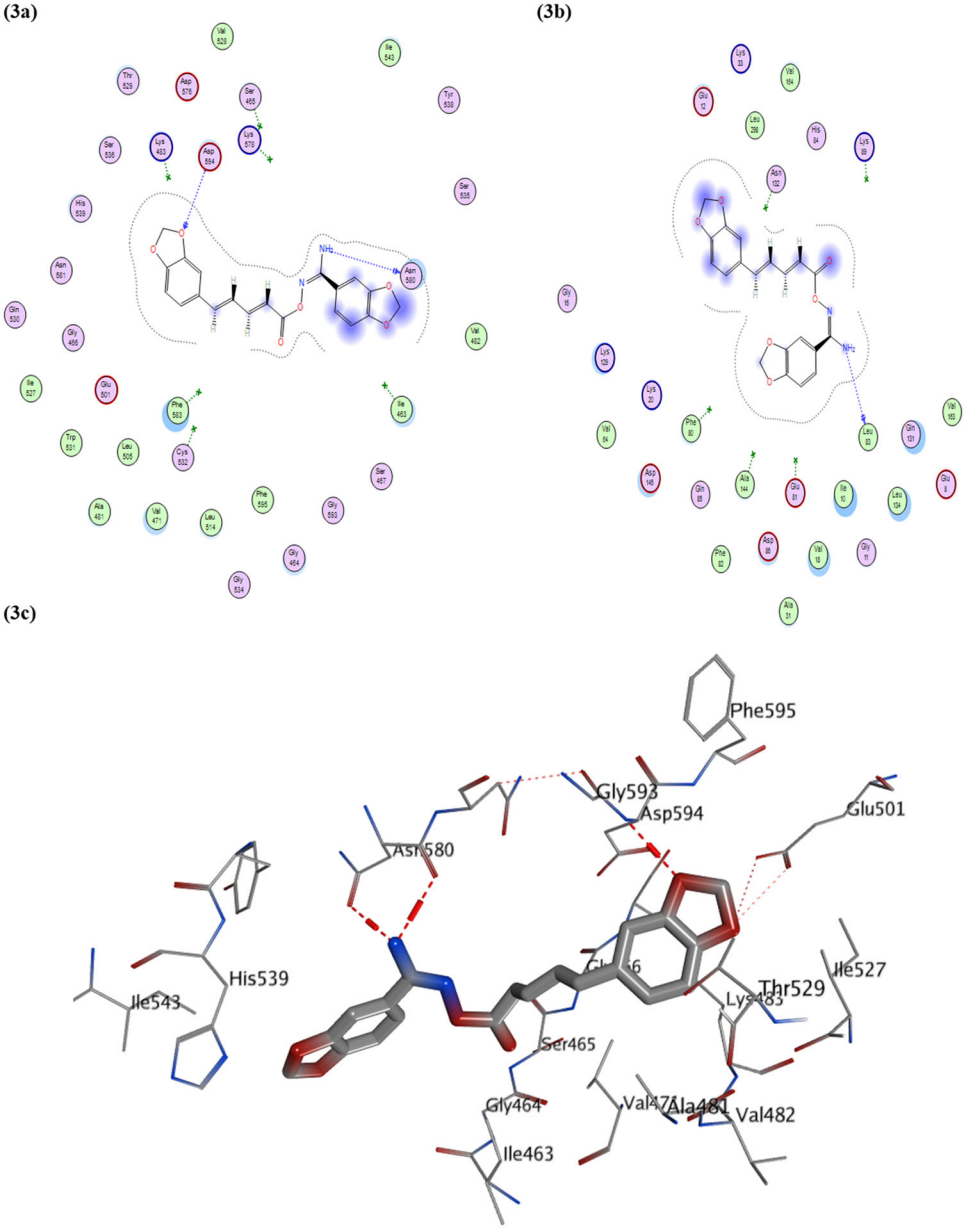
Schematic representation of docking interactions of compound **VIk** within both BRAF^V600E^ (PDB ID: 4MNF) and CDK2 (PDB ID: 1PYE): (a,b) 2 D Interactions of **VIk** within 4MNF and 1PYE; respectively; (c) Presumptive binding modes of **VIk** within 4MNF active site as 3 D diagram: showing three H-bond interactions with Asn 580 and Asp 594 coloured in red-colour‎.

As shown in Figure 3(a), compound **VIk** managed to have three strong binding interactions in the form of H-bond donor and acceptor bonds, which stabilised its structure within the active site of BRAF^V600E^ kinase protein, which was likely also seen with compound **VIf** (as shown in Figure S2), confirming how such class of compound could inhibit BRAF^V600E^ protein.

On the other hand, visual inspections of the best docking poses obtained with molecular docking of both compounds **VIf** and **VIk** within active site of CDK2 protein, revealed their achievement in having H-bonding interactions with two key amino acid residues GLU81 and LEU83 as did the co-crystallised ligand (as shown in Figure 3(b,c) and Figure S3). Additionally, compound **VIc** showed two H-bonding interactions with another two amino acid residues (ASN 132 and LYS89, as shown in Figure S3) rather than key amino acid residues, and this could explain its lower potency compared to its congers **VIf** and **VIk** against CDK2 protein.

To summarise, molecular docking simulations revealed a good docking score of such class of compounds within three active sites used in this study, as well as a good idea about the mode of inhibition of these compounds within BRAF^V600E^ and CDK2 active sites.

### ADME parameters and drug-likeness computational analysis

From our efforts to investigate bioavailability of such class of compounds, we measured physicochemical descriptors, pharmacokinetics, drug-like nature, and medicinal chemistry friendliness of all newly synthesised 11 compounds via SwissADME free web tool^34^.

Starting with role of five (Lipinski’s RO5)^36^^,^^37^, all newly synthesised derivatives **VIa-k** have values for physicochemical within required ranges of candidate drugs, as shown in Table S2 (supporting information**)**. Briefly, having H-bonding centres (either acceptor or donor) helps in enhancing water solubility and H-bond formation with various amino acid residues lining target active sites, in the same way, having acceptable number of rotating bonds helps in adaptation and flexibility alignment of such molecules within target active sites. Additionally, having a partition coefficient (i.e. lipophilicity parameter) within −0.5 to ≤ 5, and both TPSA and MR values below 140 and 130; respectively, generally indicates good probability of penetration through cell membranes, gastrointestinal penetration, and hence bioavailability. Fortunately, all the newly synthesised were found to not a substantial substrate for permeability glycoprotein (which is also known as multidrug resistance protein which is responsible for efflux of drugs out of cells and hence reduce their pharmacological efficacy) and having Abbott oral bioavailability score above zero, indicating their high probability of medicinal impact and biological activities in clinical trials^38^.

Finally, all the compounds pass the five medicinal chemistry filters (Lipinski, Ghose, Veber, Egan, and Muegge) and Pan-assay of interference compounds (PAINS), indicating their specificity for target proteins affected by them and being a drug-candidate for drug discovery and development, as shown in Table S2.

## Conclusion

In the search for multi-targeted antiproliferative agents, a novel series of piperine-carboximidamide hybrids was designed and synthesised as EGFR, BRAF^V600E^, and CDK2 inhibitors. The new hybrids demonstrated promising antiproliferative properties. Some new hybrids exhibited strong EGFR and/or BRAF^V600E^ inhibitory activity. Furthermore, some potent antiproliferative derivatives outperformed the reference drug against CDK2. Docking simulations within the active sites of BRAF^V600E^ and CDK2 provided insight into the potential mode of inhibition of these compounds. Furthermore, the new class of compounds follows Lipinski’s rule of five, as well as passing all medicinal chemistry filters and having an acceptable bioavailability profile. Following optimisation, these novel hybrids could be considered as potential anticancer agents.

## Materials and methods

### Chemistry

General Details: See Appendix A

#### Plant material

Black Pepper seeds were purchased from the local market and grounded to a powder then kept in dry container. They were ground to 40 mesh and stored in airtight container until further use.

#### Extraction, isolation and purification of piperine alkaloid from black Pepper

##### Extraction

The sample of black pepper (100 g) was refluxed with 300 ml of dichloromethane for 2 h. in a round bottom flask^23^. Attach a water condenser to the top of the flask and allow water to run through it to condense the CH_2_Cl_2_ vapours. After cooling the flask, use vacuum filtration with a Büchner funnel and filter paper to filter out the pepper grounds. Wash the grounds with 50 ml CH_2_Cl_2_. The filtrate resulting from the extraction solutions were concentrated to dryness using rotary evaporator until a dark brown oil is left.

##### Thin layer chromatography

Thin layer chromatography experiment were carried out on commercial silica coated plates using hexane: ethyl acetate 7:3 (v/v) as a mobile phase (R*_f_* = 0.36 for piperine)

##### Preparative silica gel chromatography

Adsorption of the extract on activated silica gel. Prepared sample of the extract was subjected to a silica gel column (200 g) and eluted with hexane: ethyl acetate system in a gradient manner (increasing polarity) from 15 to 40%. Fractions were collected and monitored by TLC method using hexane: ethyl acetate 7:3 (v/v) as a solvent system and concentrated H_2_SO_4_ was used as spraying reagent to identify those fractions containing the target compound.

##### Crystallisation of piperine

The piperine-containing fractions identified by TLC were pooled and the volatiles removed under reduced pressure using rotary evaporator to provide a thick orange oil placed in an ice bath for cooling. The mass of the thus obtained material, being highly enriched in piperine. 50 ml of cold diethyl ether was added with continuous stirring. After stirring for 5 min, the solvent was evaporated again by using rotary evaporator. The oil was placed in an ice bath for it to cool down. 50 ml of cold diethyl ether was added again and left in the refrigerator for about 24 h. After 24 h, the yellowish piperine precipitates were collected and washed with small volumes of cold diethyl ether. Two more re-crystallisation steps were carried out to rise the purity of the piperine.

Compounds **II** and **Va-k** were prepared as previously described^24^,^25^.

#### General method for the synthesis of compounds via-k

The *N,N'*-carbonyl diimidazole CDI (2.29 mmol, 2eq.) was added to a stirred solution of piperic acid **(II)** (1.14 mmol, 1eq.) in dry acetonitrile (10 ml) and stirred for 1 h at room temperature. The appropriate amidoximes **Va-l** were then added to the resulting mixture and stirred for another 10 h. Following the completion of the reaction (monitored by TLC), The precipitate **VIa-k** that developed was filtered and then crystallised from acetonitrile.

##### *N*'-{[(*2E,4E*)-5-(2*H*-1,3-Benzodioxol-5-yl)penta-2,4-dienoyl]oxy}benzene carboximidamide (via)

Yield: 0.27 g (70%), yellow solid, m.p: 175–177 °C, *R_f_*: 0.26 (Hexane: Ethyl acetate, 2:1, v/v); ^1^H NMR (400 MHz, *δ* ppm DMSO-*d*_6_): 7.73 (d, *J* = 7.2 Hz, 2H, Ar-H), 7.55–7.44 (m, 5H, Ar-H), 7.29 (s, 1H, Ar-H), 7.04–7.02 (m, 4H, CH = CH), 6.87 (s, broad, 2H, NH_2_), 6.06 (s, 2H, O-CH_2_-O); ^13^C NMR (100 MHz, *δ* ppm DMSO-*d*_6_): 164.35, 156.49, 148.23, 148.01, 144.78, 140.34, 131.79, 130.52, 128.35, 126.76, 124.99, 123.29, 118.96, 108.53, 105.77, 101.41. Anal. Calc. (%) for C_19_H_16_N_2_O_4_: C, 67.85; H, 4.80; N, 8.33; Found: C, 67.86; H, 4.87; N, 8.46.

##### *N*'-{[(*2E,4E*)-5-(2*H*-1,3-Benzodioxol-5-yl)penta-2,4-dienoyl]oxy}-4-chlorobenzene-1-carboximidamide (VIb)

Yield: 0.08 g (35%), yellow solid, m.p: 157–160 °C, *R_f_.* 0.28 (Hexane: Ethyl acetate, 2:1, v/v); ^1^H NMR (400 MHz, *δ* ppm DMSO-*d*_6_): 8.55 (s, 1H, Ar-H), 7.82 (d, *J* = 8.6 Hz, 2H, Ar-H), 7.72–7.65 (m, 3H, Ar-H), 7.45 (d, *J* = 8.5 Hz, 1H, Ar-H), 7.25 (t, *J* = 7.7 Hz, 2H, CH = CH), 7.14–7.06 (m, 2H, CH = CH), 7.1 (s, broad, NH_2_), 6.09 (s, 2H, O-CH_2_-O); ^13^C NMR (100 MHz, *δ* ppm DMSO-*d*_6_): 162.00, 156.64, 152.26, 149.18, 148.85, 148.13, 143.62, 136.88, 135.88, 130.59, 129.99, 129.34, 128.64, 128.53, 128.12, 127.83, 127.15, 124.51, 124.02, 117.78, 116.64, 108.73, 105.90, 101.60. Anal. Calc. (%) for C_19_H_15_ClN_2_O_4_: C, 61.55; H, 4.08; N, 7.56. Found: C, 61.86; H, 4.20; N, 7.77.

##### *N*'-{[(*2E,4E*)-5-(2*H*-1,3-Benzodioxol-5-yl)penta-2,4-dienoyl]oxy}-4-bromobenzene-1-carboximidamide (VIc)

Yield: 0.12 g (42.1%), yellow solid, m.p: 159–161 °C, *R_f_*: 0.29 (Hexane: Ethyl acetate, 2:1, v/v); IR (FTIR): ν_max_ (cm^−1^) 3491,3358 (NH_2_), 3134, 2919 (CH), 1769 (C = O), 1624, 1590 (C = N and C = C), 834 (Ar-CH bending); ^1^H NMR (400 MHz, *δ* ppm DMSO-*d*_6_): 8.55 (s, 1H, Ar-H), 7.82 (s, 1H, Ar-H), 7.82–7.74 (m, 1H, Ar-H), 7.26 (t, *J* = 7.27 Hz, 2H, CH = CH), 7.14–7.08 (m, 4H, Ar-H), 7.00 (s, 2H, CH = CH), 6.97 (s, broad, 2H, NH_2_), 6.09 (s, 2H, O-CH_2_-O). ^13^C NMR (100 MHz, *δ* ppm DMSO-*d*_6)_ : 161.99, 156.67, 152.27, 149.13, 148.86, 148.14, 143.59, 136.92, 132.15, 131.45, 131.02, 130.37, 130.10, 128.88, 127.96, 127.38, 124.49, 124.11, 117.80, 116.63, 108.72, 105.91, 101.59. Anal. Calc. (%) for C_19_H_15_BrN_2_O_4_: C, 54.96; H, 3.64; N, 6.75. Found: C, 55.12; H, 3.75; N, 6.87.

##### *N*'-{[(*2E,4E*)-5-(2*H*-1,3-Benzodioxol-5-yl)penta-2,4-dienoyl]oxy}-4-methoxybenzene-1-carboximidamide (VId)

Yield: 0.24 g (72.7%), yellow solid, m.p: 145–148 °C, *R_f_*: 0.29 (Hexane: Ethyl acetate, 2:1, v/v); IR (FTIR): ν_max_ (cm^−1^) 3498,3381 (NH_2_), 3134, 2978 (CH), 1760 (C = O), 1683, 1588 (C = N and C = C), 827 (Ar-CH bending); ^1^H NMR (400 MHz, *δ* ppm DMSO-*d*_6_): 8.55 (s, 1H, Ar-H), 7.82 (s, 1H, Ar-H), 7.77 (d, *J* = 10.9 Hz, 1H, Ar-H), 7.69 (d, *J* = 8.5 Hz, 2H, Ar-H), 7.14–7.06 (m, 4H, CH = CH), 7.00 (d, *J* = 8.7 Hz, 2H, Ar-H), 6.76 (s, broad, 2H, NH_2_), 6.09 (s, 2H, O-CH_2_-O), 3.8 (s, 3H, O-CH_3_). ^13^C NMR (100 MHz, *δ* ppm DMSO-*d*_6)_ : 161.50, 157.61, 152.98, 149.64, 144.08, 128.68, 124.99, 124.43, 123.68, 118.23, 117.20, 114.23, 109.22, 106.35, 102.10, 55.84. Anal. Calc. (%) for C_20_H_18_N_2_O_5_: C, 65.57; H, 4.95; N, 7.65. Found: C, 65.43; H, 5.10; N, 7.77.

##### *N*'-{[(*2E,4E*)-5-(2*H*-1,3-Benzodioxol-5-yl)penta-2,4-dienoyl]oxy}-4-methylbenzene-1-carboximidamide (VIe)

Yield: 0.20 g (50%), yellow solid, m.p: 185–188 °C, *R_f_*: 0.24 (Hexane: Ethyl acetate, 2:1, v/v); ^1^H NMR (400 MHz, *δ* ppm DMSO-*d*_6_): 7.63 (d, *J* = 7.76 Hz, 2H, Ar-H), 7.53–7.46 (m, 1H, CH = CH), 7.28 (s, 1H, Ar-H), 7.25 (d, *J* = 7.76 Hz, 2H, Ar-H), 7.00 (d, *J* = 4.36 Hz, 2H, CH = CH) , 7.00 (d, *J* = 4.36 Hz, 1H, Ar-H) , 6.93 (d, *J* = 7.96 Hz, 1H, Ar-H), 6.81 (s, broad, 2H, NH_2_), 6.18 (d, *J* = 15.4 Hz, 1H, CH = CH), 6.00 (s, 2H, O-CH_2_-O), 2.50 (s, 3H, Ar-CH_3_). ^13^C NMR (100 MHz, *δ* ppm DMSO-*d*_6)_ : 164.40, 156.35, 148.22, 148.06, 144.70, 140.28, 140.15, 130.50, 128.86, 126.65, 125.00, 123.29, 119.01, 108.52, 105.78, 101.40, 20.94. Anal. Calc. (%) for C_20_H_18_N_2_O_4_: C, 68.56; H, 5.18; N, 8.00. Found: C, 68.80; H, 5.30; N, 8.17.

##### *N*'-{[(2E,4E)-5-(2*H*-1,3-benzodioxol-5-yl)penta-2,4-dienoyl]oxy}-2-chlorobenzene-1-carboximidamide (VIf)

Yield: 0.134 g (63%), yellow solid, m.p: 165–167 °C, *R_f_*: 0.26 (Hexane: Ethyl acetate, 2:1, v/v); IR (FTIR): ν_max_ (cm^−1^) 3491,3358 (NH_2_), 3134, 2919 (CH), 1769 (C = O), 1624, 1590 (C = N and C = C), 834 (Ar-CH bending); ^1^H NMR (400 MHz, *δ* ppm DMSO-*d*_6_): 8.54 (s, 1H, Ar-H), 7.57–7.47 (m, 4H, Ar-H), 7.43 (d, *J* = 7.56 Hz, 1H, Ar-H), 7.26 (d, *J* = 8.2 Hz, 1H, Ar-H), 7.25 (d, *J* = 15.4 Hz, 1H, CH = CH) , 7.14–7.07 (m, 3H, CH = CH), 6.97 (s, broad, 2H, NH_2_), 6.08 (s, 2H, O-CH_2_-O). ^13^C NMR (100 MHz, *δ* ppm DMSO-*d*_6)_ : 162.03, 156.68, 152.22, 149.17, 148.84, 148.13, 143.57, 136.92, 132.12, 131.12, 130.50, 129.89, 129.50, 126.97, 124.44, 123.57, 117.99, 116.46, 108.79, 105.87, 101.42. Anal. Calc. (%) for C_19_H_15_ClN_2_O_4_: C, 61.55; H, 4.08; N, 7.56. Found: C, 61.67; H, 4.23; N, 7.77.

##### *N*'-{[(2E,4E)-5-(2*H*-1,3-benzodioxol-5-yl)penta-2,4-dienoyl]oxy}-3-chlorobenzene-1-carboximidamide (VIg)

Yield: 0.08 g (44.4%), yellow solid, m.p: 163–165 °C, *R_f_*: 0.26 (Hexane: Ethyl acetate, 2:1, v/v); ^1^H NMR (400 MHz, *δ* ppm DMSO-*d*_6_): 8.28 (s, 1H, Ar-H), 7.65–7.59 (m, 2H, Ar-H), 7.15–7.06 (m,2H, CH = CH), 7.08 (s, broad, 2H, NH_2_), 6.95 (d, *J* = 7.6 Hz, 1H, Ar-H), 6.08 (s, 2H, O-CH_2_-O). ^13^C NMR (100 MHz, *δ* ppm DMSO-*d*_6)_ :, 162.33, 149.95, 149.35, 144.26, 130.96, 130.66, 124.96, 124.36, 118.28, 117.06, 109.70, 106.28, 101.87, Anal. Calc. (%) for C_19_H_15_ClN_2_O_4_: C, 61.55; H, 4.08; N, 7.56. Found: C, 61.43; H, 4.26; N, 7.83.

##### *N*'-{[(2E,4E)-5-(2*H*-1,3-benzodioxol-5-yl)penta-2,4-dienoyl]oxy}-3-bromobenzene-1-carboximidamide (VIh)

Yield: 0.34 g (72.34%), yellow solid, m.p: 141–143 °C, *R_f_*: 0.28 (Hexane: Ethyl acetate, 2:1, v/v); IR (FTIR): ν_max_ (cm^−1^) 3486, 3348 (NH_2_), 3134, 2916 (CH), 1778 (C = O), 1622, 1589 (C = N and C = C), 988, 830, 761 (Ar-CH bending); ^1^H NMR (400 MHz, *δ* ppm DMSO-*d*_6_): 8.55 (s, 1H, Ar-H), 7.92 (s, 2H, Ar-H), 7.75 (t, *J* = 6.74 Hz, 4H, Ar-H), 7.45 (t, *J* = 7.9 Hz, 1H, Ar-H), 7.45 (t, *J* = 7.9 Hz, 1H, CH = CH), 7.27 (t, *J* = 7.7 Hz, 1H, Ar-H), 7.27 (t, *J* = 7.7 Hz, 1H, CH = CH) , 7.13–7.07 (m, 2H, CH = CH), 6.98 (s, broad, 2H, NH2), 6.09 (s, 2H, O-CH_2_-O). ^13^C NMR (100 MHz, *δ* ppm DMSO-*d*_6)_ : 162.33, 156.64, 152.46, 149.65, 148.36, 143.95, 133.85, 131.27, 130.36, 129.67, 126.25, 124.96, 124.36, 122.08, 118.28, 117.37, 109.17, 106.28, 101.87. Anal. Calc. (%) for C_19_H_15_BrN_2_O_4_: C, 54.96; H, 3.64; N, 6.75. Found: C, 55.06; H, 3.79; N, 6.97.

##### *N*'-{[(2E,4E)-5-(2*H*-1,3-benzodioxol-5-yl)penta-2,4-dienoyl]oxy}-3,4-dimethoxybenzene-1-carboximidamide (VIi)

Yield: 0.134 g (37.2%), yellow solid, m.p: 147–149 °C, *R_f_*: 0.24 (Hexane: Ethyl acetate, 2:1, v/v); IR (FTIR): ν_max_ (cm^−1^) 3490, 3378 (NH_2_), 3079, 2938 (CH), 1757 (C = O), 1623, 1585 (C = N and C = C), 766 (Ar-CH bending); ^1^H NMR (400 MHz, *δ* ppm DMSO-*d*_6_): 8.56 (s, 1H, Ar-H), 7.36–7.29 (m, 4H, Ar-H), 7.25 (d, *J* = 7.33 Hz, 1H, Ar-H), 7.15–7.08 (m, 2H, CH = CH), 7.03 (d, *J* = 8.34 Hz, 2H, CH = CH), 6.81 (s, broad, 2H, NH2), 6.09 (s, 2H, O-CH_2_-O), 3.81 (s, 6H, -(OCH_3_)_2)_ . ^13^C NMR (100 MHz, *δ* ppm DMSO-*d*_6)_ : 162.14, 158.69, 157.38, 152.55, 151.32, 150.63, 149.40, 148.71, 148.10, 125.69, 123.16, 119.63, 117.71, 116.79, 111.95, 111.03, 110.04, 109.12, 55.70 . Anal. Calc. (%) for C_21_H_20_N_2_O_6_: C, 63.63; H, 5.09; N, 7.07. Found: C, 63.50; H, 5.29; N, 7.17.

##### *N*'-{[(2E,4E)-5-(2*H*-1,3-benzodioxol-5-yl)penta-2,4-dienoyl]oxy} naphthalene -1-carboximidamide (VIj)

Yield: 0.148 g (70%), yellow solid, m.p: 170–172 °C, *R_f_*: 0.26 (Hexane: Ethyl acetate, 2:1, v/v); ^1^H NMR (400 MHz, *δ* ppm DMSO-*d*_6_): 8.56 (s, 1H, Ar-H), 8.00 (m, 2H, Ar-H), 7.59 (m, 5H, Ar-H), 7.31 (s, 1H, Ar-H), 7.07 (m, 3H, CH = CH), 7.05 (s, broad, 2H, NH2), 6.95 (d, *J* = 8.0 Hz, 1H, Ar-H), 6.22 (d, *J* = 15.3 Hz, 1H, CH = CH), 6.08 (s, 2H, O-CH_2_-O). ^13^C NMR (100 MHz, *δ* ppm DMSO-*d*_6)_ : 164.84, 157.55, 148.66, 145.25, 140.76, 133.47, 131.27, 130.05, 128.46, 127.47, 127.17, 126.56, 126.25, 125.57, 123.98, 119.57, 109.02, 106.25, 101.87 . Anal. Calc. (%) for C_23_H_18_N_2_O_4_: C, 71.49; H, 4.70; N, 7.25. Found: C, 71.55; H, 4.83; N, 7.47.

##### *N*'-{[(*2E,4E*)-5-(2*H*-1,3-Benzodioxol-5-yl)penta-2,4-dienoyl]oxy}-2*H*-naphtho[1,2-d][1,3]dioxole-8-carboximidamide (VIk)

Yield: 0.09 g (70%), yellow solid, m.p: 166–168 °C, *R_f_*: 0.27 (Hexane: Ethyl acetate, 2:1, v/v); ^1^H NMR (400 MHz, *δ* ppm DMSO-*d*_6_): 8.41 (s, 1H, Ar-H), 7.79 (s, 1H, Ar-H), 7.4–7.3 (m, 2H, Ar-H), 7.00 (s, broad, 2H, NH2), 6.95(dd, *J* = 8.3 Hz, 7.7 Hz, 2H, Ar-H), 6.74–6.82 (m, 2H, CH = CH), 6.68 (d, *J* = 8 Hz, 1H, CH = CH), 6.19 (d, *J* = 15.4 Hz, 1H, CH = CH), 6.06 (s, 2H, O-CH_2_-O), 5.93 (s, 2H, O-CH_2_-O). ^13^C NMR (100 MHz, *δ* ppm DMSO-*d*_6)_ : 164.46, 162.16, 158.71, 156.40, 150.04, 149.12, 147.81, 147.20, 144.59, 141.44, 140.44, 135.00, 130.54, 125.40, 125.01, 123.18, 121.57, 120.95, 119.03, 118.04, 110.36, 108.83, 108.14, 107.83, 105.60, 102.07, 101.58, 100.77 . Anal. Calc. (%) for C_24_H_18_N_2_O_6_: C, 66.97; H, 4.22; N, 6.51. Found: C, 67.17; H, 4.40; N, 6.65.

### Biology

#### Cell viability testing and IC_50_ determination

##### MTT assay

The MTT assay was used to assess how the synthetic compounds impacted the viability of mammary epithelial cells (MCF-10A)^26^^,^^27^. Appendix A.

##### Antiproliferative test

The MTT assay was performed using various cell lines in accordance with previously reported procedures^28^^,^^29^ to investigate the antiproliferative potential of **VIa-k**. Refer to Appendix A.

##### EGFR inhibitory assay

The EGFR-TK test^30^ was used to evaluate the inhibitory potency of the most potent derivatives **VIc**, **VIf**, **VIg**, **VIi**, and **VIk** against EGFR. See Appendix A.

##### BRAF kinase assay

An *in vitro* study^31^ was conducted to evaluate the anti-BRAF^V600E^ of **VIc**, **VIf**, **VIg**, **VIi**, and **Vik**. See Appendix A.

##### CDK2-TK assay

Compounds **VIc**, **VIf**, **VIg**, **VIi**, and **VIk** were further investigated for their potential to inhibit the CDK2 enzyme^32^. See Appendix A.

##### LOX-IMVI melanoma cell line cytotoxicity assay

The MTT cytotoxicity assay was used to assess the anticancer activity of **VIc**, **VIf**, and **VIk** on the LOX-IMVI melanoma cell line, which comprises BRAF^V600E^ kinase overexpression^33^. Refer to Appendix A.

### In-Silico molecular docking simulations

Molecular Operating Environment (MOE® 2014.0901) software was used to evaluate and examine possible interactions of test molecules (**VIc**, **VIf**, **VIg**, **VIi**, and **VIk**) within crystal structure of 3 target proteins: Epidermal growth factor receptor (EGFR; PDB ID: 1M17), BRAF^V600E^ kinase (PDB ID: 4MNF) and cyclin-dependent kinase (CDK2; PDB ID: 1PYE) obtained from RSCB protein data bank^34^ and results in the form of docking score (S; kcal/mol), docking accuracy expressed as root-mean square deviation (RMSD; Å) and binding interactions with various amino acid residues lining active site (as listed in Table S1). Preparation of structural formulas of test molecules and structure of target proteins were performed as reported elsewhere^35^. Validation of prepared protein structures was done via re-docking of co-crystallised ligands with their protein crystal-structure obtained from RSCB protein data bank and their docking score and RMSD values were within acceptable range for running docking simulations within target proteins (as listed in Table S1). Docking simulations were performed as docking protocol reported elsewhere and results were reported in Table S1^35^. Visual inspection of produced docking poses (10 poses/molecule) for binding interactions with various amino acid residues lining active site of both 4MNF and 1PYE crystal structure and were listed in Table S1 and represented as 2 D and 3 D diagrams in Figures 1–2, Figures S1–S3.

### ADME parameters and drug-likeness computational analysis

SwissADME free web tool for prediction of physicochemical parameters^34^, pharmacokinetics, and drug-likeness of small molecules^,^ was used to measure such parameters of compounds **VIa**-**VIk** and results were listed in Table S2. Also, the medicinal chemistry filters (Lipinski, Ghose, Veber, Egan, and Muegge) were applied on all test compounds and none disobey such filters. PAINS (Pan-Assay of Interference Compounds) were used to examine specificity of such class of compounds as possible anticancer agents and all compounds gave zero score as shown in Table S2.

## Supplementary Material

Supplemental Material

## References

[1] Zha G-F, Qin H-L, Youssif BGM, Amjad MW, Raja MAG, Abdelazeem AH, Bukhari SNA. Discovery of potential anticancer multi-targeted ligustrazine based cyclohexanone and oxime analogs overcoming the cancer multidrug resistance. Eur J Med Chem. 2017;135:34–48. 28431353 10.1016/j.ejmech.2017.04.025

[2] Hisham M, Youssif BGM, Osman EEA, Hayallah AM, Abdel-Aziz M. Synthesis and biological evaluation of novel xanthine derivatives as potential apoptotic antitumor agents. Eur J Med Chem. 2019;176:117–128. 31108261 10.1016/j.ejmech.2019.05.015

[3] Youssif BGM, Abdelrahman MH, Abdelazeem AH, Abdelgawad MA, Ibrahim HM, Salem OIA, Mohamed MFA, Treambleau L, Bukhari SNA. Design, synthesis, mechanistic and histopathological studies of small-molecules of novel indole-2-carboxamides and pyrazino[1,2-a]indol-1(2*H*)-ones as potential anticancer agents effecting the reactive oxygen species production. Eur J Med Chem. 2018;146:260–273. 29407956 10.1016/j.ejmech.2018.01.042

[4] Abou-Zied HA, Youssif BGM, Mohamed MFA, Hayallah AM, Abdel-Aziz M. Design, synthesis, anticancer activity and docking studies of novel xanthine derivatives carrying chalcone moiety as hybrid molecules. Bioorg Chem. 2019;89:102997. 31136902 10.1016/j.bioorg.2019.102997

[5] El-Sherief HAM, Youssif BGM, Abbas Bukhari SN, Abdelazeem AH, Abdel-Aziz M, Abdel-Rahman HM. Synthesis, anticancer activity and molecular modeling studies of 1,2,4-triazole derivatives as EGFR inhibitors. Eur J Med Chem. 2018;156:774–789. 30055463 10.1016/j.ejmech.2018.07.024

[6] Hughes D, Andersson DI. Evolutionary consequences of drug resistance: shared principles across diverse targets and organisms. Nat Rev Genet. 2015;16(8):459–471. 26149714 10.1038/nrg3922

[7] Lehár J, Krueger AS, Avery W, Heilbut AM, Johansen LM, Price ER, Rickles RJ, Short GF, Staunton JE, Jin X, et al. Synergistic drug combinations tend to improve therapeutically relevant selectivity. Nat Biotechnol. 2009;27(7):659–666. 19581876 10.1038/nbt.1549 PMC2708317

[8] Shah KN, Bhatt R, Rotow J, Rohrberg J, Olivas V, Wang VE, Hemmati G, Martins MM, Maynard A, Kuhn J, et al. Aurora kinase A drives the evolution of resistance to third-generation EGFR inhibitors in lung cancer. Nat Med. 2019;25(1):111–118. 30478424 10.1038/s41591-018-0264-7 PMC6324945

[9] Umadevi P, Deepti D, Venugopal DVR. Synthesis, anticancer and antibacterial activities of piperine analogs. Med Chem Res. 2013;22(11):5466–5471.

[10] Deng Y, Sriwiriyajan S, Tedasen A, Hiransai P, Graidist P. Anti-cancer effects of Piper nigrum via inducing multiple molecular signaling *in vivo* and *in vitro*. J Ethnopharmacol. 2016;188:87–95. 27155135 10.1016/j.jep.2016.04.047

[11] Katz L, Baltz RH. Natural product discovery: past, present, and future. J Ind Microbiol Biotechnol. 2016;43(2–3):155–176. 26739136 10.1007/s10295-015-1723-5

[12] Newman DJ, Cragg GM, Snader KM. Natural products as sources of new drugs over the period 1981-2002. J Nat Prod. 2003;66(7):1022–1037. 12880330 10.1021/np030096l

[13] Ali Y, Alam MS, Hamid H, Husain A, Bano S, Dhulap A, Kharbanda C, Nazreen S, Haider S. Design, synthesis, and biological evaluation of piperic acid triazolyl derivatives as potent anti-inflammatory agent. Eur J Med Chem. 2015;92:490–500. 25596479 10.1016/j.ejmech.2015.01.001

[14] Tantawy AH, Meng X-G, Marzouk AA, Fouad A, Abdelazeem AH, Youssif BGM, Jiang H, Wang M-Q. Structure-based design, synthesis, and biological evaluation of novel piperine–resveratrol hybrids as antiproliferative agents targeting SIRT-2. RSC Adv. 2021;11(41):25738–25751. 35478872 10.1039/d1ra04061h PMC9037111

[15] Baraldi PG, Bovero A, Fruttarolo F, Preti D, Tabrizi MA, Pavani MG, Romagnoli R. DNA minor groove binders as potential antitumor and antimicrobial agents. Med Res Rev. 2004;24(4):475–528. 15170593 10.1002/med.20000

[16] Karaaslan C. Synthesis and structure elucidation of new benzimidazole amidoxime derivatives. Turk J of Pharm Sci. 2020;17(1):108–114. 32454768 10.4274/tjps.galenos.2019.44270 PMC7227876

[17] Gobis K, Foks H, Kȩdzia A, et al. Studies on pyrazine derivatives. XLVII. Synthesis and antibacterial activity of novel pyrazine derivatives with amidoxime moiety. Acta Pol Pharm - Drug Res. 2006;63:39–45. 17515328

[18] Huang M-R, Hsu Y-L, Lin T-C, Cheng T-J, Li L-W, Tseng Y-W, Chou Y-S, Liu J-H, Pan S-H, Fang J-M, et al. Structure-guided development of purine amide, hydroxamate, and amidoxime for the inhibition of non-small cell lung cancer. Eur J Med Chem. 2019;181:111551. 31376567 10.1016/j.ejmech.2019.07.054

[19] Ningaiah S, Bhadraiah UK, Keshavamurthy S, Javarasetty C. Novel pyrazoline amidoxime and their 1, 2, 4-oxadiazole analogues: synthesis and pharmacological screening. Bioorg Med Chem Lett. 2013;23(16):4532–4539. 23850201 10.1016/j.bmcl.2013.06.042

[20] Burgaud JL, Ongini E, Del Soldato P. Nitric oxide-releasing drugs: a novel class of effective and safe therapeutic agents. Ann N Y Acad Sci. 2002; 962(1):360–371. 12076987 10.1111/j.1749-6632.2002.tb04080.x

[21] Youssif BGM, Gouda AM, Moustafa AH, Abdelhamid AA, Gomaa HAM, Kamal I, Marzouk AA. Design and synthesis of new triarylimidazole derivatives as dual inhibitors of BRAF^V600E^/p38α with potential antiproliferative activity. J Mol Struct. 2022;1253:132218.

[22] Obaid Arhema Frejat F, Zhai H, Cao Y, Wang L, Mostafa YA, Gomaa HAM, Youssif BGM, Wu C. Novel indazole derivatives as potent apoptotic antiproliferative agents by multi-targeted mechanism: synthesis and biological evaluation. Bioorg Chem. 2022;126:105922. 35667253 10.1016/j.bioorg.2022.105922

[23] Shingate PN, Dongre PP, Kannur DM. New method development for extraction and isolation of piperine from black pepper. IJPSR. 2013;4:3165–3170.

[24] Wang Y, Yao Y, Liu J, Wu L, Liu T, Cui J, Lee DY-W. Synthesis and biological activity of piperine derivatives as potential PPARγ agonists. Drug Des Devel Ther. 2020;14:2069–2078. 10.2147/DDDT.S238245 PMC7266110 32546971

[25] Youssif BGM, Mohamed MFA, Al-Sanea MM, Moustafa AH, Abdelhamid AA, Gomaa HAM. Novel aryl carboximidamide and 3-aryl-1,2,4-oxadiazole analogues of naproxen as dual selective COX-2/15-LOX inhibitors: design, synthesis and docking studies. Bioorg Chem. 2019;85:577–584. 30878890 10.1016/j.bioorg.2019.02.043

[26] Abdelbaset MS, Abdel-Aziz M, Abuo-Rahma GEA et al. Novel quinoline derivatives carrying nitrones/oximes nitric oxide donors: design, synthesis, antiproliferative and caspase-3 activation activities. Arch Pharm. 2019;352:1800270. 10.1002/ardp.201800270 30500087

[27] Al-Wahaibi LH, Gouda AM, Abou-Ghadir OF, Salem OIA, Ali AT, Farghaly HS, Abdelrahman MH, Trembleau L, Abdu-Allah HHM, Youssif BGM, et al. Design and synthesis of novel 2,3-dihydropyrazino[1,2-a]indole-1,4-dione derivatives as antiproliferative EGFR and BRAFV600E dual inhibitors. Bioorg Chem. 2020;104:104260. 32920363 10.1016/j.bioorg.2020.104260

[28] Mohassab AM, Hassan HA, Abdelhamid D, Gouda AM, Youssif BGM, Tateishi H, Fujita M, Otsuka M, Abdel-Aziz M. Design and synthesis of novel quinoline/chalcone/1,2,4-triazole hybrids as potent antiproliferative agent targeting EGFR and BRAF^V600E^ kinases. Bioorg Chem. 2021;106:104510. 33279248 10.1016/j.bioorg.2020.104510

[29] Marzouk AA, Abdel-Aziz SA, Abdelrahman KS, Wanas AS, Gouda AM, Youssif BGM, Abdel-Aziz M. Design and synthesis of new 1,6-dihydropyrimidin-2-thio derivatives targeting VEGFR-2: molecular docking and antiproliferative evaluation. Bioorg Chem. 2020;102:104090. 32683176 10.1016/j.bioorg.2020.104090

[30] Mohamed FAM, Gomaa HAM, Hendawy OM, Ali AT, Farghaly HS, Gouda AM, Abdelazeem AH, Abdelrahman MH, Trembleau L, Youssif BGM, et al. Design, synthesis, and biological evaluation of novel EGFR inhibitors containing 5-chloro-3-hydroxymethyl-indole-2-carboxamide scaffold with apoptotic antiproliferative activity. Bioorg Chem. 2021;112:104960. 34020242 10.1016/j.bioorg.2021.104960

[31] El-Sherief HAM, Youssif BGM, Bukhari SNA, Abdel-Aziz M, Abdel-Rahman HM. Novel 1,2,4-triazole derivatives as potential anticancer agents: design, synthesis, molecular docking and mechanistic studies. Bioorg Chem. 2018;76:314–325. 29227915 10.1016/j.bioorg.2017.12.013

[32] Mekheimer RA, Allam SMR, Al-Sheikh MA, Moustafa MS, Al-Mousawi SM, Mostafa YA, Youssif BGM, Gomaa HAM, Hayallah AM, Abdelaziz M, et al. Discovery of new pyrimido[5,4-c]quinolines as potential antiproliferative agents with multitarget actions: rapid synthesis, docking, and ADME studies. Bioorg Chem. 2022;121:105693. 35219045 10.1016/j.bioorg.2022.105693

[33] Gomaa HAM, Shaker ME, Alzarea SI, Hendawy OM, Mohamed FAM, Gouda AM, Ali AT, Morcoss MM, Abdelrahman MH, Trembleau L, et al. Optimization and SAR investigation of novel 2,3-dihydropyrazino[1,2-a]indole-1,4-dione derivatives as EGFR and BRAF^V600E^ dual inhibitors with potent antiproliferative and antioxidant activities. Bioorg Chem. 2022;120:105616. 35078049 10.1016/j.bioorg.2022.105616

[34] Daina A, Michielin O, Zoete V. SwissADME: a free web tool to evaluate pharmacokinetics, drug-likeness and medicinal chemistry friendliness of small molecules. Sci Rep. 2017;7(1):42717. 10.1038/srep42717 PMC5335600 28256516

[35] Abdel-Wahab NM, Gomaa AA, Mostafa YA, et al. Diterpenoids profile of the marine sponge Chelonaplysilla erecta and candidacy as potential ‎antitumor drugs investigated by molecular docking and pharmacokinetic studies. Nat Prod ‎Res. 2022;36(20):1–5. 10.1080/14786419.2022.2063856 35400256

[36] Lipinski CA. Drug-like properties and the causes of poor solubility and poor permeability. J Pharmacol Toxicol Methods. 2000;44(1):235–249. 11274893 10.1016/s1056-8719(00)00107-6

[37] Lipinski CA, Lombardo F, Dominy BW, Feeney PJ. Experimental and computational approaches to estimate solubility and permeability in drug discovery and development settings. Adv. Drug Delivery ‎Rev. 1997;23(1-3):3–25. 10.1016/s0169-409x(00)00129-0 11259830

[38] Martin YC. A bioavailability score. J Med Chem. 2005;48(9):3164–3170. 15857122 10.1021/jm0492002

