# Supplementary material for: Novel piperine-carboximidamide hybrids: design, synthesis, and antiproliferative activity via a multi-targeted inhibitory pathway
Source: J Enzyme Inhib Med Chem. 2022 Nov 30;38(1):376–86. doi: 10.1080/14756366.2022.2151593 (PMC9721426; doi:10.1080/14756366.2022.2151593)
Supplement: Supplemental Material [file IENZ_A_2151593_SM0905.pdf]

Novel piperine-carboximidamide hybrids: design, synthesis, and antiproliferative activity via a multi-kinases' inhibitory pathway

Supplementary Data

^{1}H NMR spectrum of (VIa) :

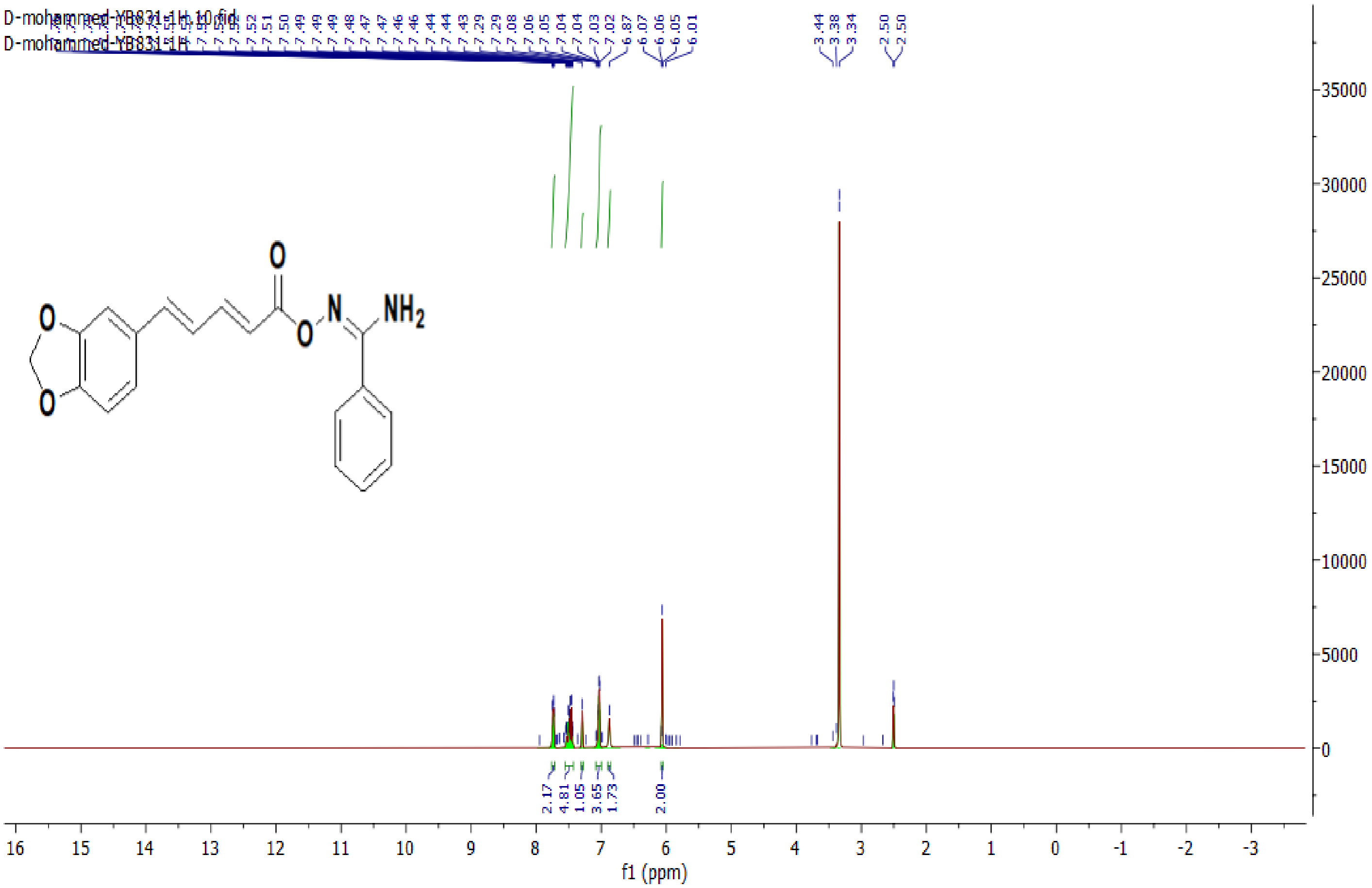

^{1}H NMR (400 MHz, δ ppm DMSO-d_6): 7.73 (d, J = 7.2 Hz, 2H, Ar-H), 7.55-7.44 (m, 5H, Ar-H) , 7.29 (s, 1H, Ar-H), 7.04-7.02 (m, 4H, CH=CH), 6.87 (s, broad, 2H, NH_2), 6.06 (s, 2H, O-CH_2-O)

Information Classification: General

^{13}C NMR spectrum of (VIa):

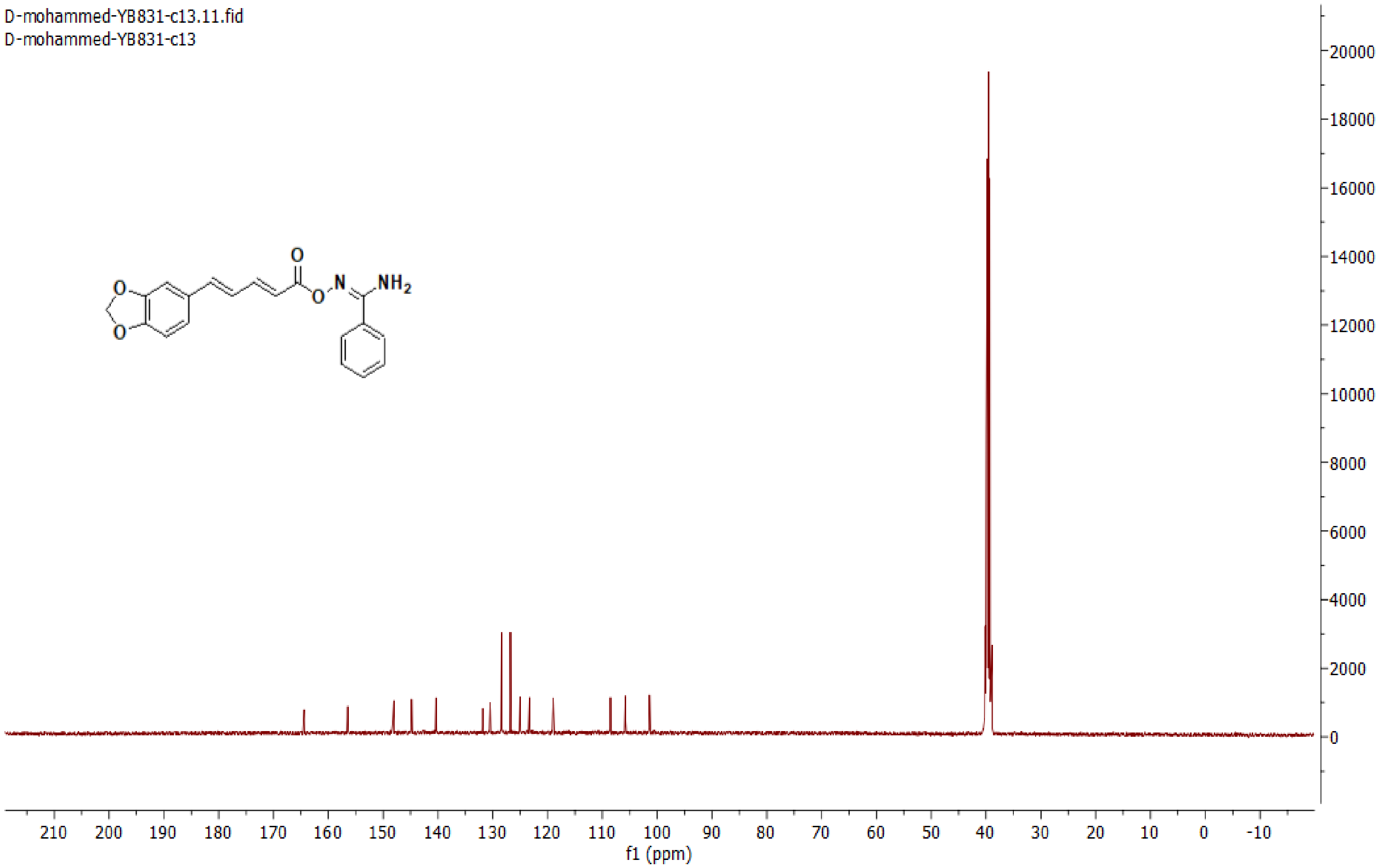

^{13}C NMR (100 MHz, δ ppm DMSO-d_6): 164.35, 156.49, 148.23, 148.01, 144.78, 140.34, 131.79, 130.52, 128.35, 126.76, 124.99, 123.29, 118.96, 108.53, 105.77, 101.41

^{1}H NMR spectrum of (VIb) :

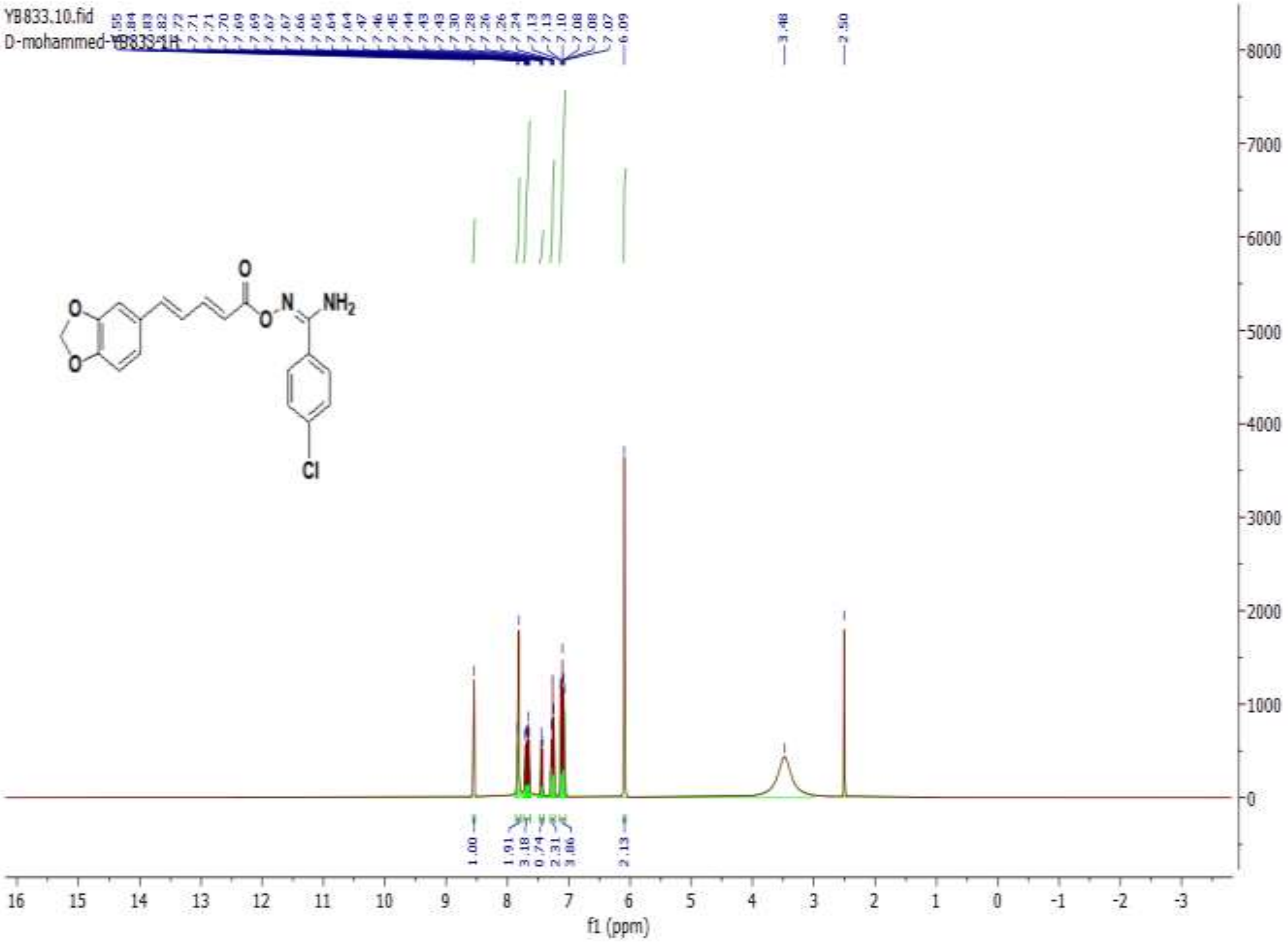

^{1}H NMR (400 MHz, δ ppm DMSO-d_6): 8.55 (s, 1H, Ar-H), 7.82 (d, J = 8.6 Hz, 2H, Ar-H), 7.72-7.65 (m, 3H, Ar-H), 7.45 (d, J = 8.5 Hz, 1H, Ar-H), 7.25 (t, J = 7.7 Hz, 2H, CH=CH), 7.14-7.06 (m, 2H, CH=CH), 7.1 (s, broad, NH_2), 6.09 (s, 2H, O-CH_2-O)

^{13}C NMR spectrum of (VIb):

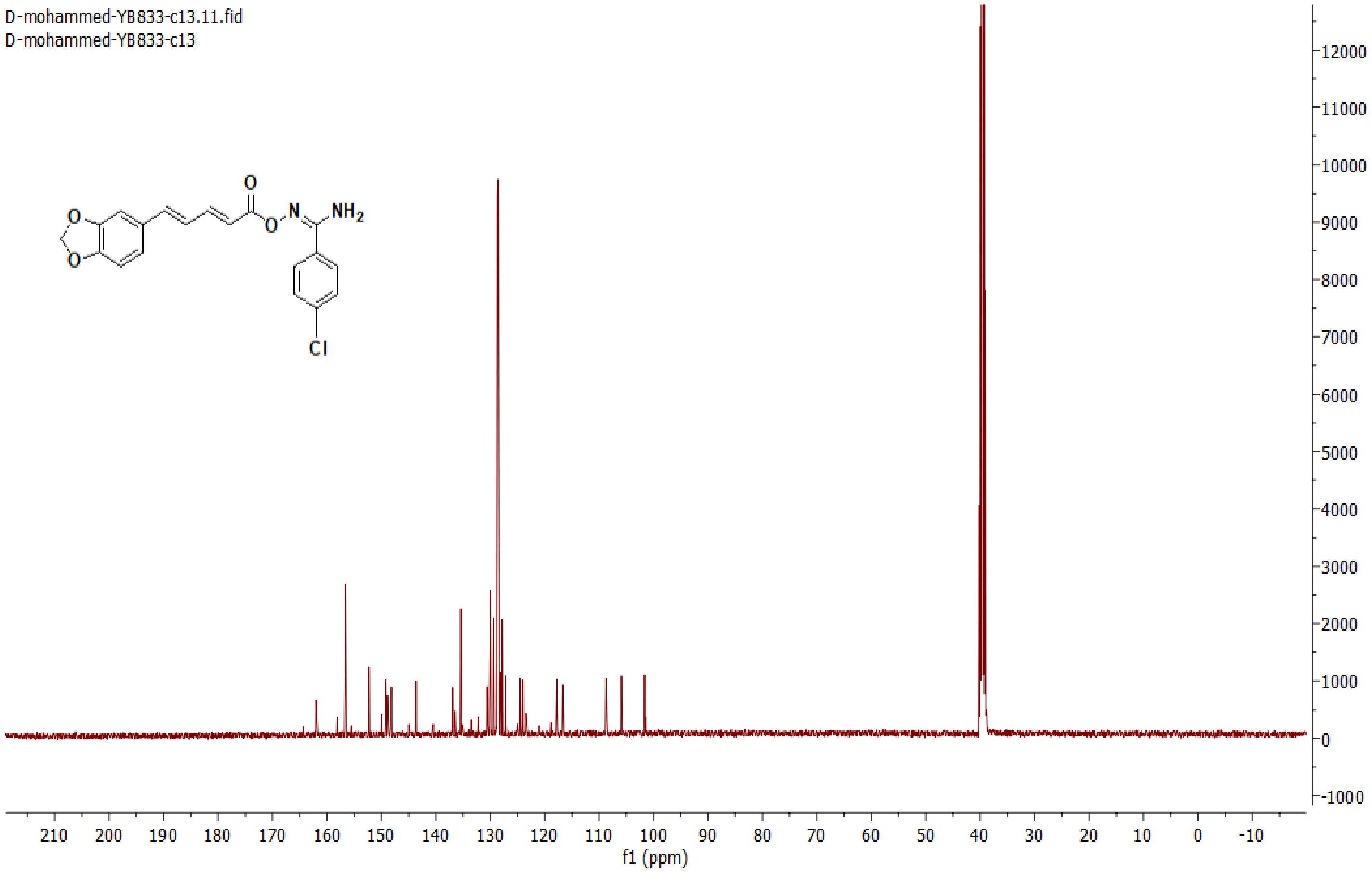

^{13}C NMR (100 MHz, δ ppm DMSO-d_6): 162.00, 156.64, 152.26, 149.18, 148.85, 148.13, 143.62, 136.88, 135.88, 130.59, 129.99, 129.34, 128.64, 128.53, 128.12, 127.83, 127.15, 124.51, 124.02, 117.78, 116.64, 108.73, 105.90, 101.60

Information Classification: General

IR spectrum of (VIc):

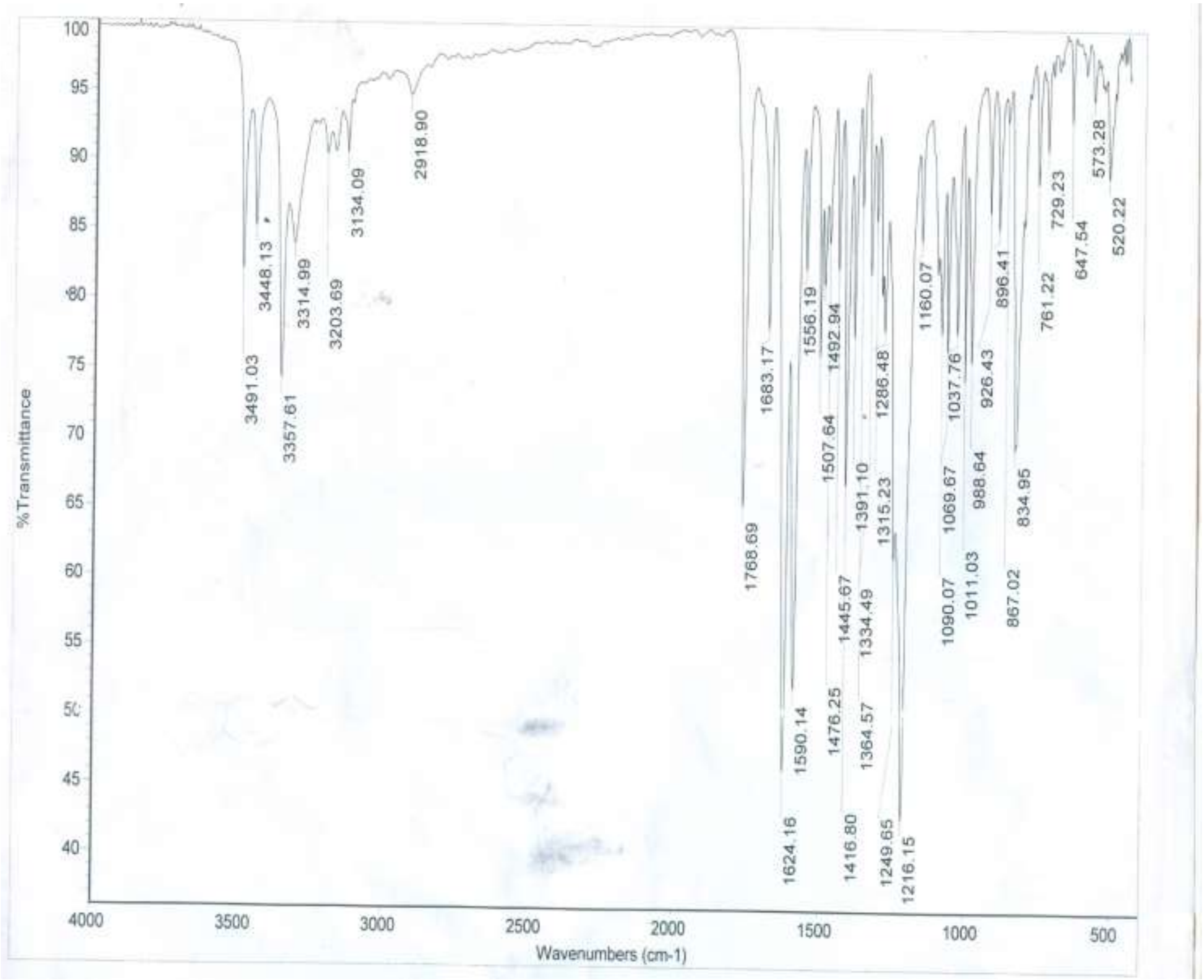

Information Classification: General

^{1}H NMR spectrum of (VIc) :

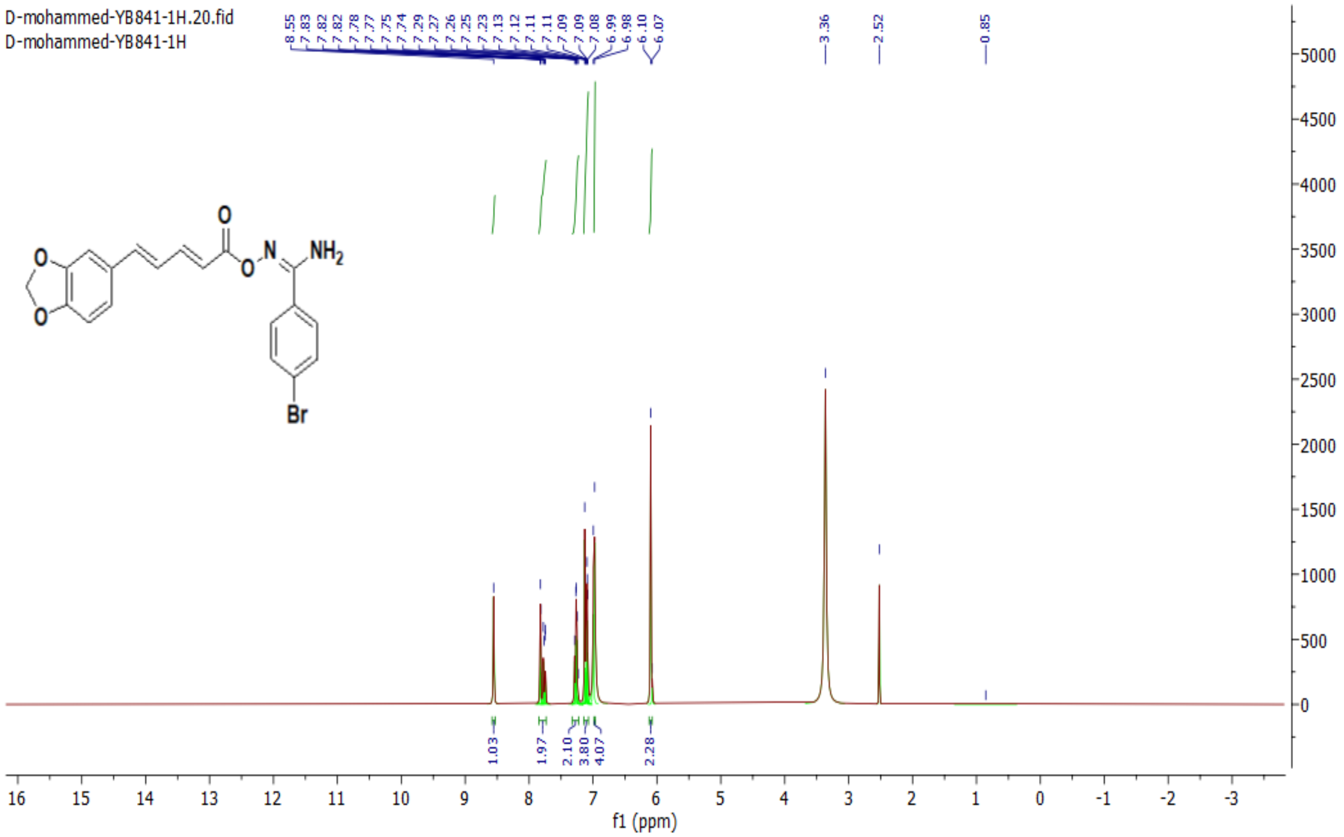

^{1}H NMR (400 MHz, δ ppm DMSO-d_6): 8.55 (s, 1H, Ar-H), 7.82 (s, 1H, Ar-H), 7.82-7.74 (m, 1H, Ar-H), 7.26 (t, J = 7.27 Hz, 2H, CH=CH), 7.14-7.08 (m, 4H, Ar-H), 7.00 (s, 2H, CH=CH), 6.97 (s, broad, 2H, NH_2), 6.09 (s, 2H, O-CH_2-O)

^{13}C NMR spectrum of (VIc) :

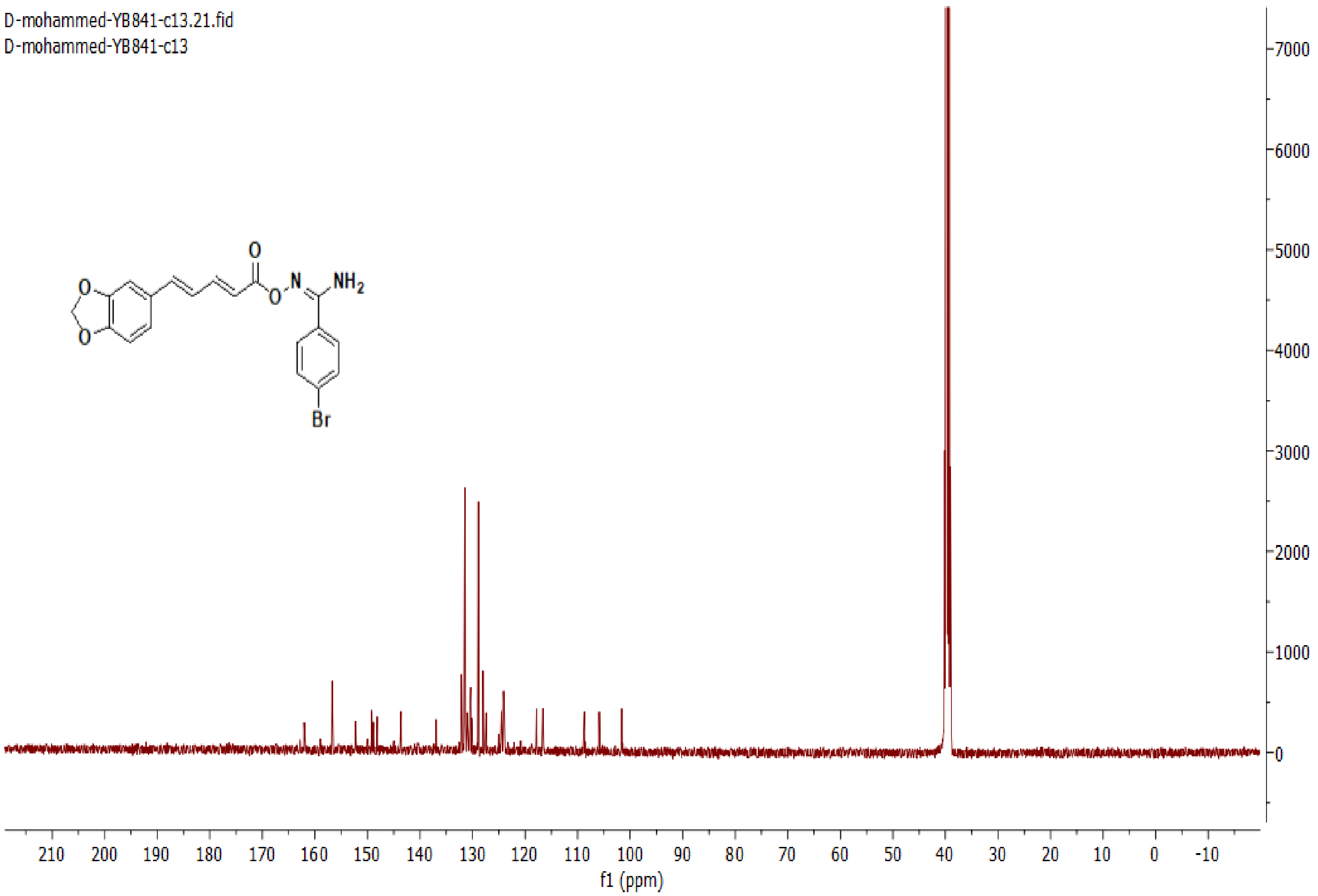

^{13}C NMR (100 MHz, δ ppm DMSO-d_6): 161.99, 156.67, 152.27, 149.13, 148.86, 148.14, 143.59, 136.92, 132.15, 131.45, 131.02, 130.37, 130.10, 128.88, 127.96, 127.38, 124.49, 124.11, 117.80, 116.63, 108.72, 105.91, 101.59

Information Classification: General

IR spectrum of (VId):

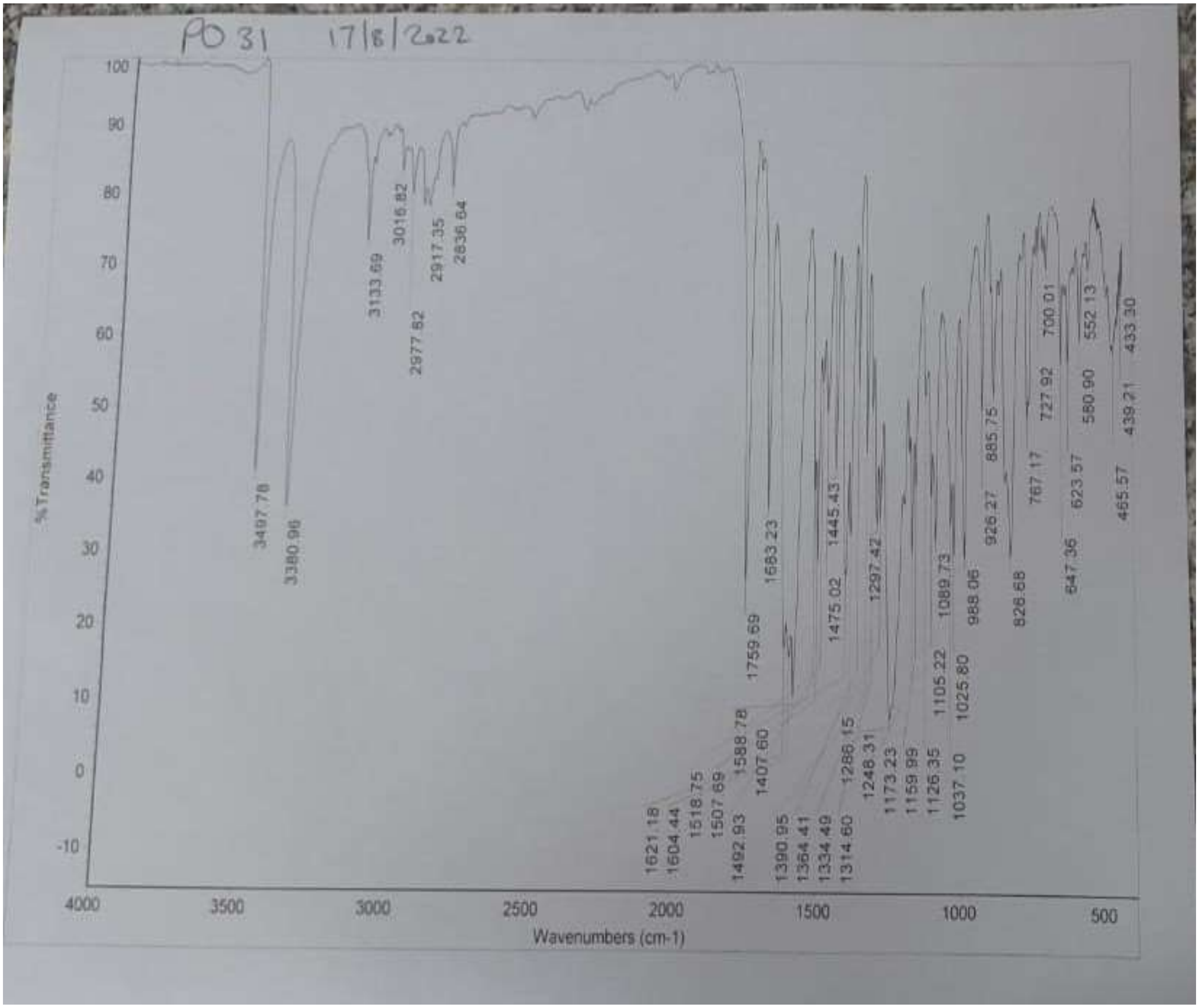

^{1}H NMR spectrum of (VId):

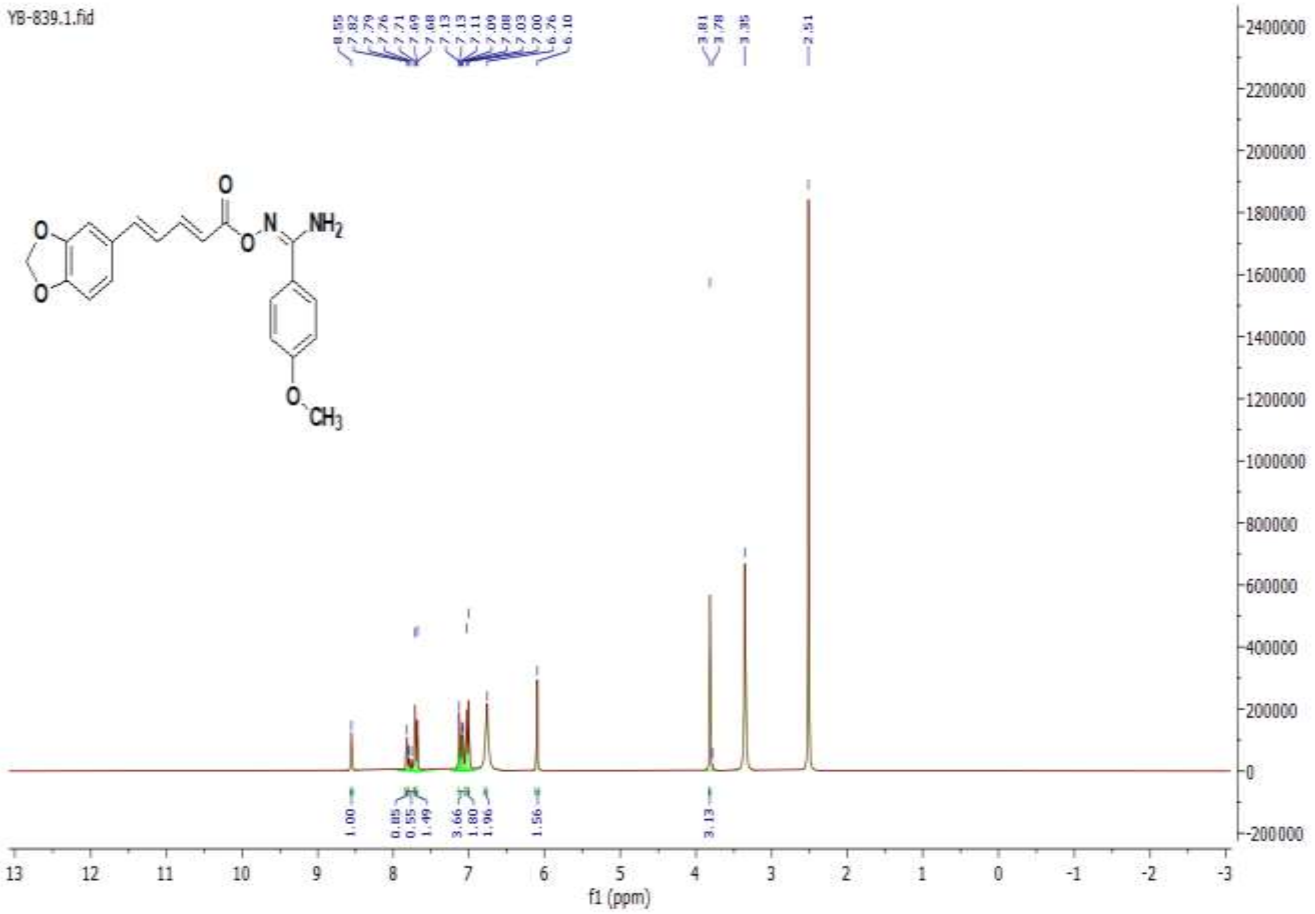

); ^{1}H NMR (400 MHz, δ ppm DMSO-d_6): 8.55 (s, 1H, Ar-H), 7.82 (s, 1H, Ar-H), 7.77 (d, J = 10.9 Hz, 1H, Ar-H), 7.69 (d, J = 8.5 Hz, 2H, Ar-H), 7.14-7.06 (m, 4H, CH=CH), 7.00 (d, J = 8.7 Hz, 2H, Ar-H), 6.76 (s, broad, 2H, NH_2), 6.09 (s, 2H, O-CH_2-O), 3.8 (s, 3H, O-CH_3)

Information Classification: General

^{13}C NMR spectrum of (VId) :

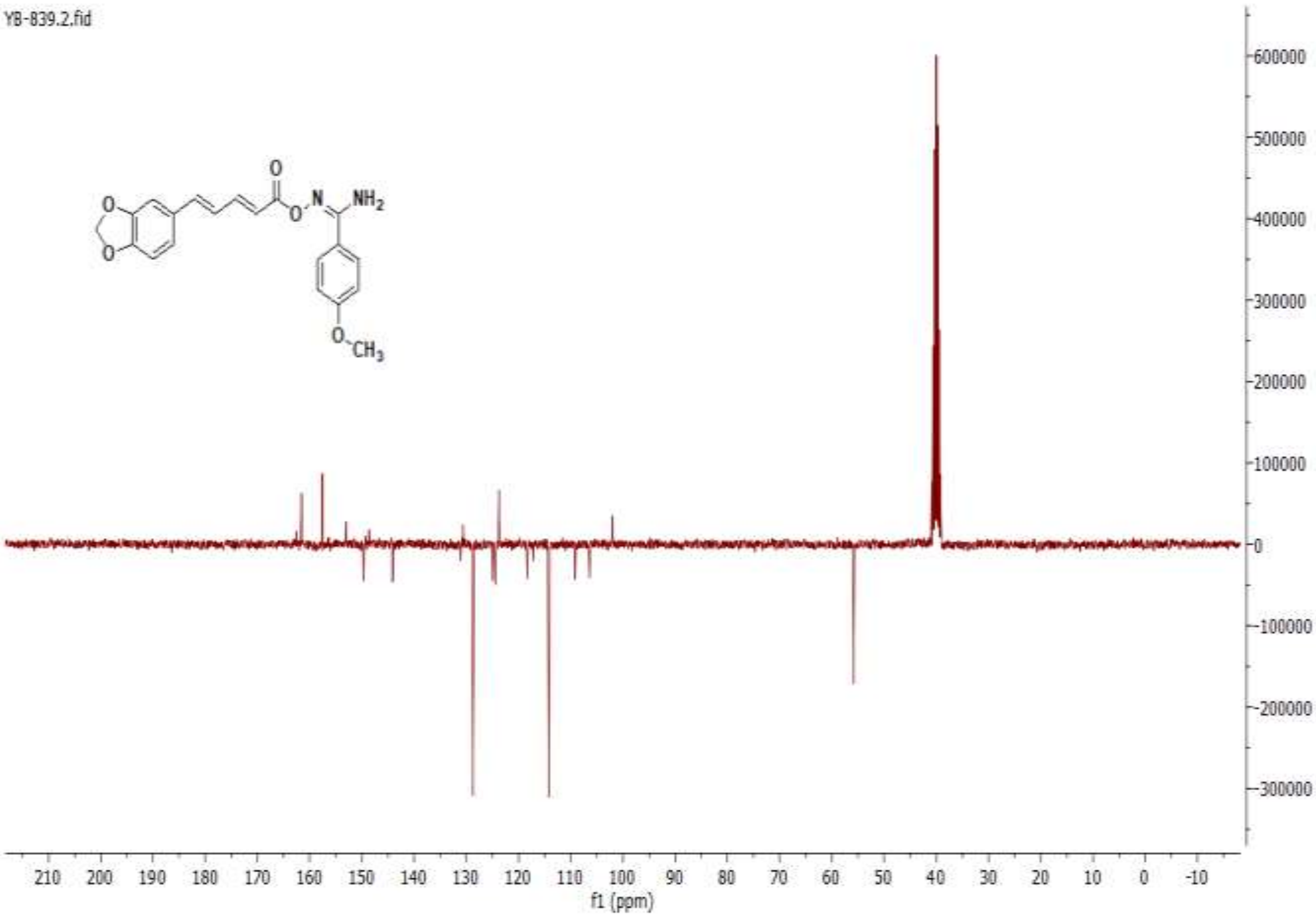

^{13}C NMR (100 MHz, δ ppm DMSO-d_6): 161.50, 157.61, 152.98, 149.64, 144.08, 128.68, 124.99, 124.43, 123.68, 118.23, 117.20, 114.23, 109.22, 106.35, 102.10, 55.84

Information Classification: General

^{1}H NMR spectrum of (VIe) :

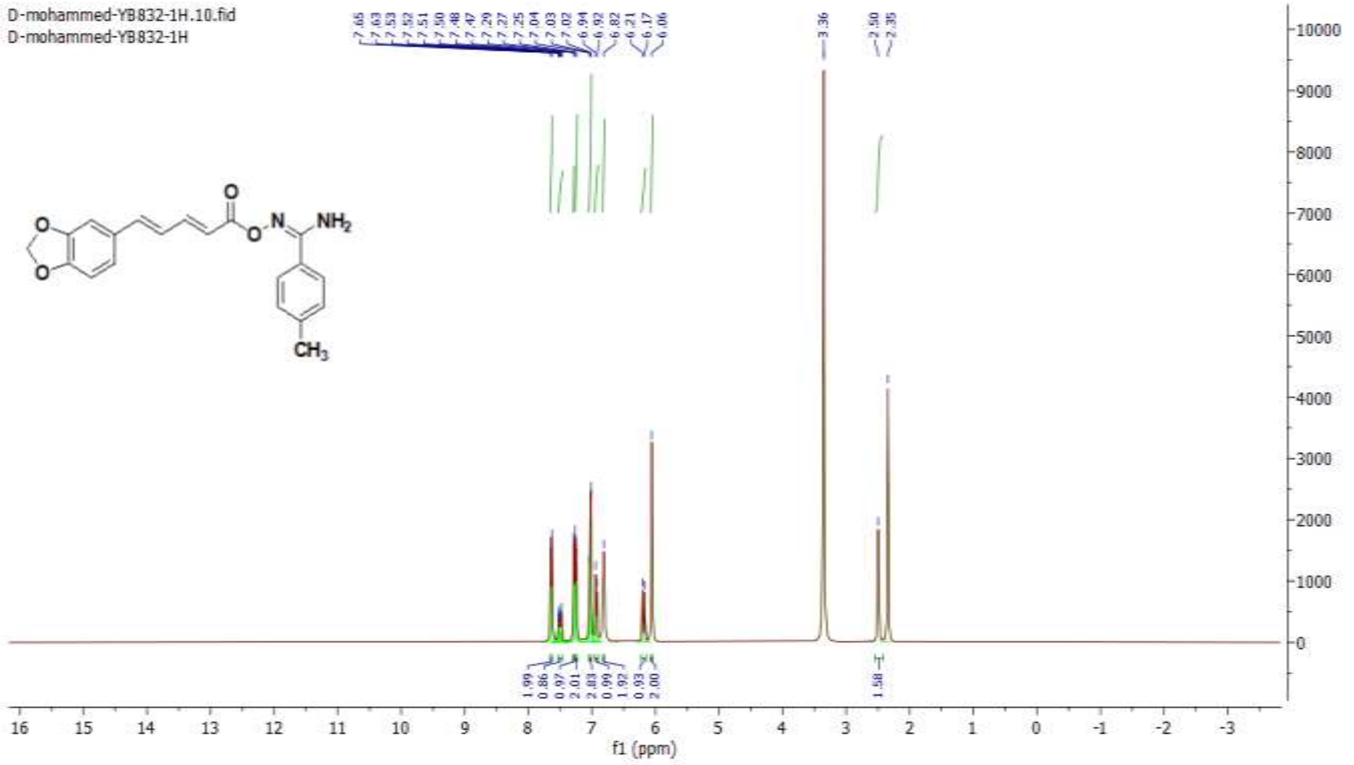

^{1}H NMR (400 MHz, δ ppm DMSO-d_6): 7.63 (d, J = 7.76 Hz, 2H, Ar-H), 7.53-7.46 (m, 1H, CH=CH), 7.28 (s, 1H, Ar-H), 7.25 (d, J = 7.76 Hz, 2H, Ar-H), 7.00 (d, J = 4.36 Hz, 2H, CH=CH), 7.00 (d, J = 4.36 Hz, 1H, Ar-H), 6.93 (d, J = 7.96 Hz, 1H, Ar-H), 6.81 (s, broad, 2H, NH_2), 6.18 (d, J = 15.4 Hz, 1H, CH=CH), 6.00 (s, 2H, O-CH_2-O), 2.50 (s, 3H , Ar-CH_3)

^{13}C NMR spectrum of (VIe) :

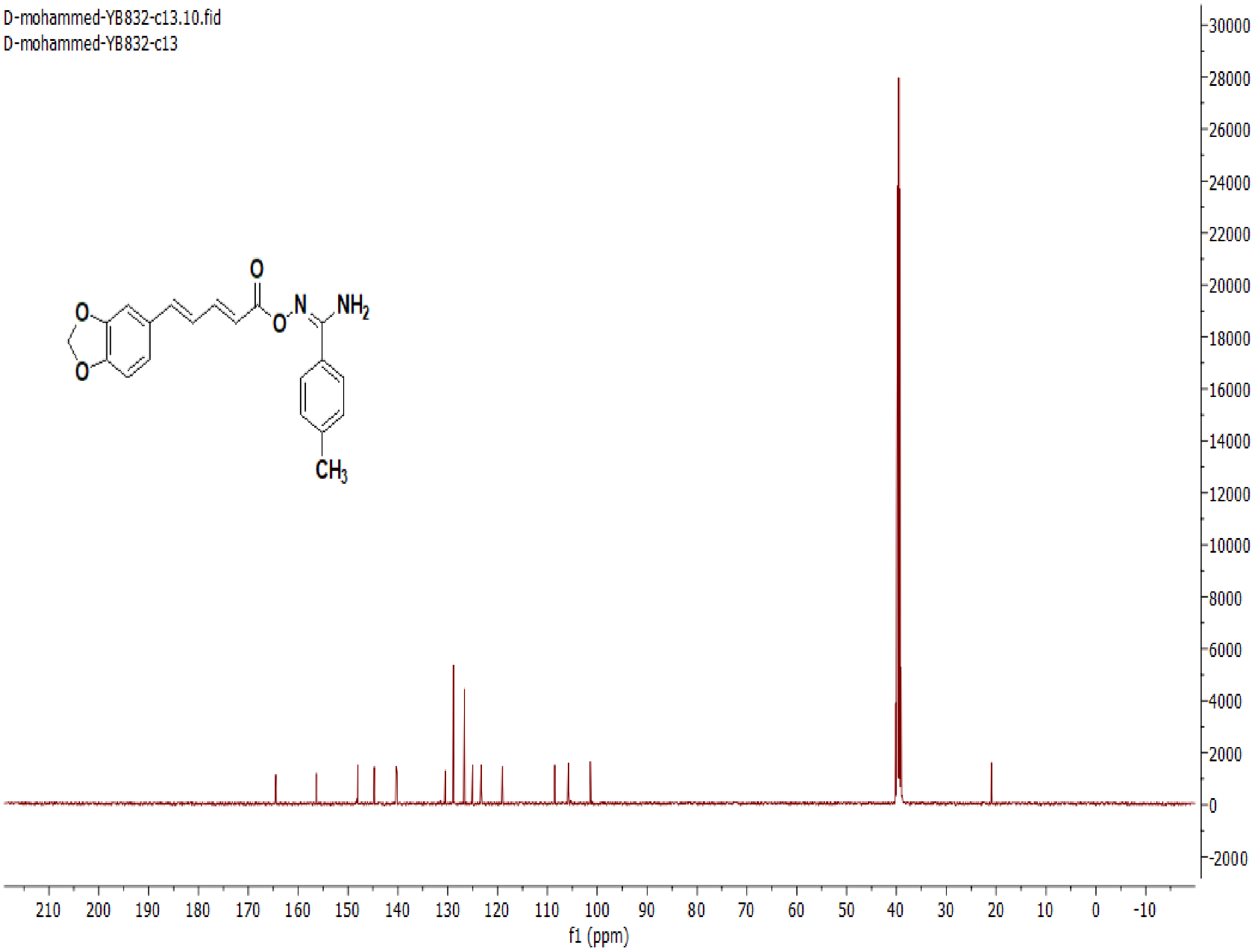

^{13}C NMR (100 MHz, δ ppm DMSO-d_6): 164.40, 156.35, 148.22, 148.06, 144.70, 140.28, 140.15, 130.50, 128.86, 126.65, 125.00, 123.29, 119.01, 108.52, 105.78, 101.40, 20.94

Information Classification: General

^{1}H NMR spectrum of (VIf) :

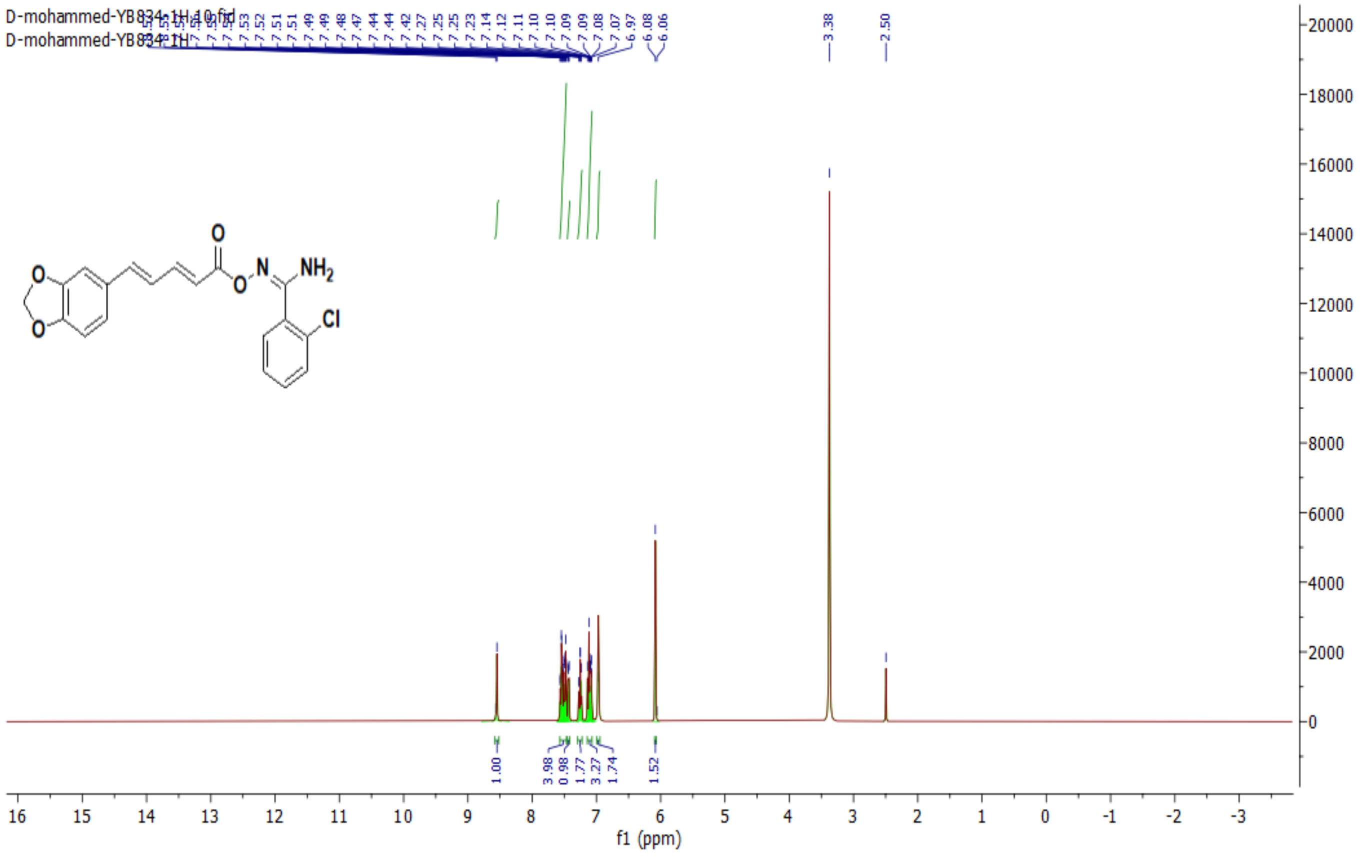

^{1}H NMR (400 MHz, δ ppm DMSO-d_6): 8.54 (s, 1H, Ar-H), 7.57-7.47 (m, 4H, Ar-H), 7.43 (d, J = 7.56 Hz, 1H, Ar-H), 7.26 (d, J = 8.2 Hz, 1H, Ar-H), 7.25 (d, J = 15.4 Hz, 1H, CH=CH), 7.14-7.07 (m, 3H, CH=CH), 6.97 (s, broad, 2H, NH_2), 6.08 (s, 2H, O-CH_2-O)

Information Classification: General

^{13}C NMR spectrum of (VIf) :

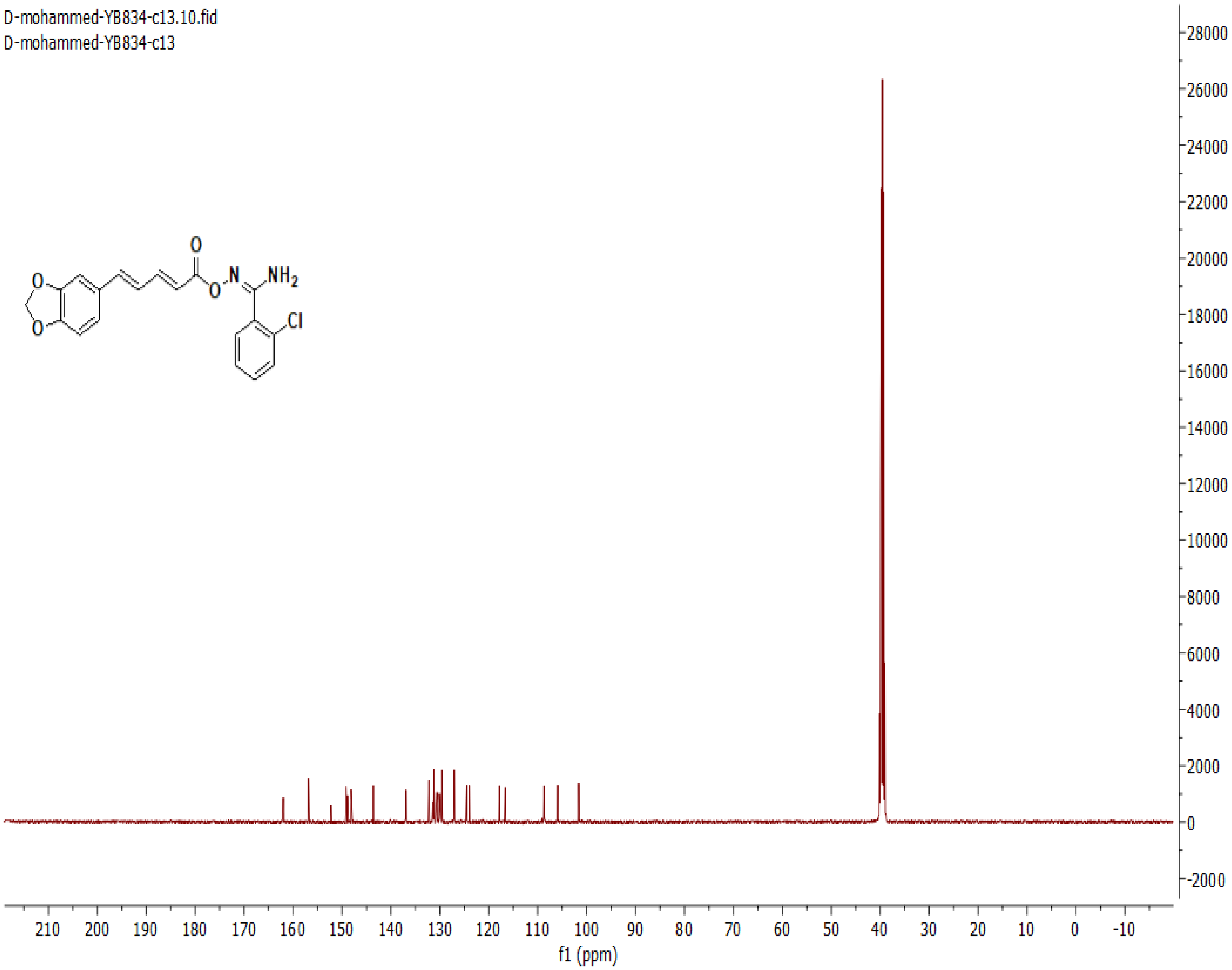

^{13}C NMR (100 MHz, δ ppm DMSO-d_6): 162.03, 156.68 , 152.22, 149.17, 148.84, 148.13, 143.57, 136.92, 132.12, 131.12, 130.50, 129.89, 129.50, 126.97, 124.44, 123.57, 117.99, 116.46, 108.79, 105.87, 101.42

Information Classification: General

^{1}H NMR spectrum of (VIg) :

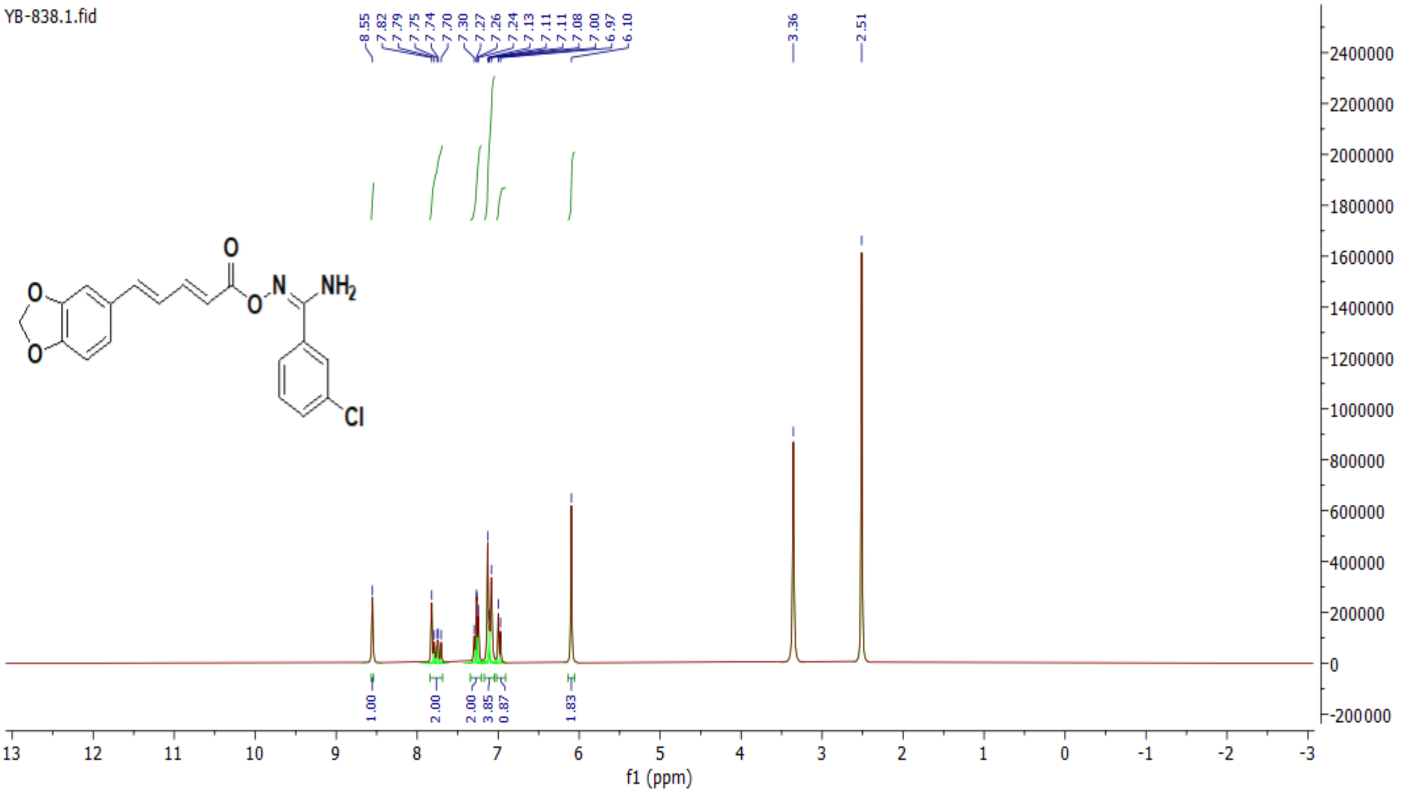

^{1}H NMR (400 MHz, δ ppm DMSO-d_6): 8.28 (s, 1H, Ar-H), 7.65-7.59 (m, 2H, Ar-H), 7.15-7.06 (m,2H, CH=CH), 7.08 (s, broad, 2H, NH_2), 6.95 (d, J = 7.6 Hz, 1H, Ar-H), 6.08 (s, 2H, O-CH_2-O)

Information Classification: General

^{13}**C NMR spectrum of (VIg) :**

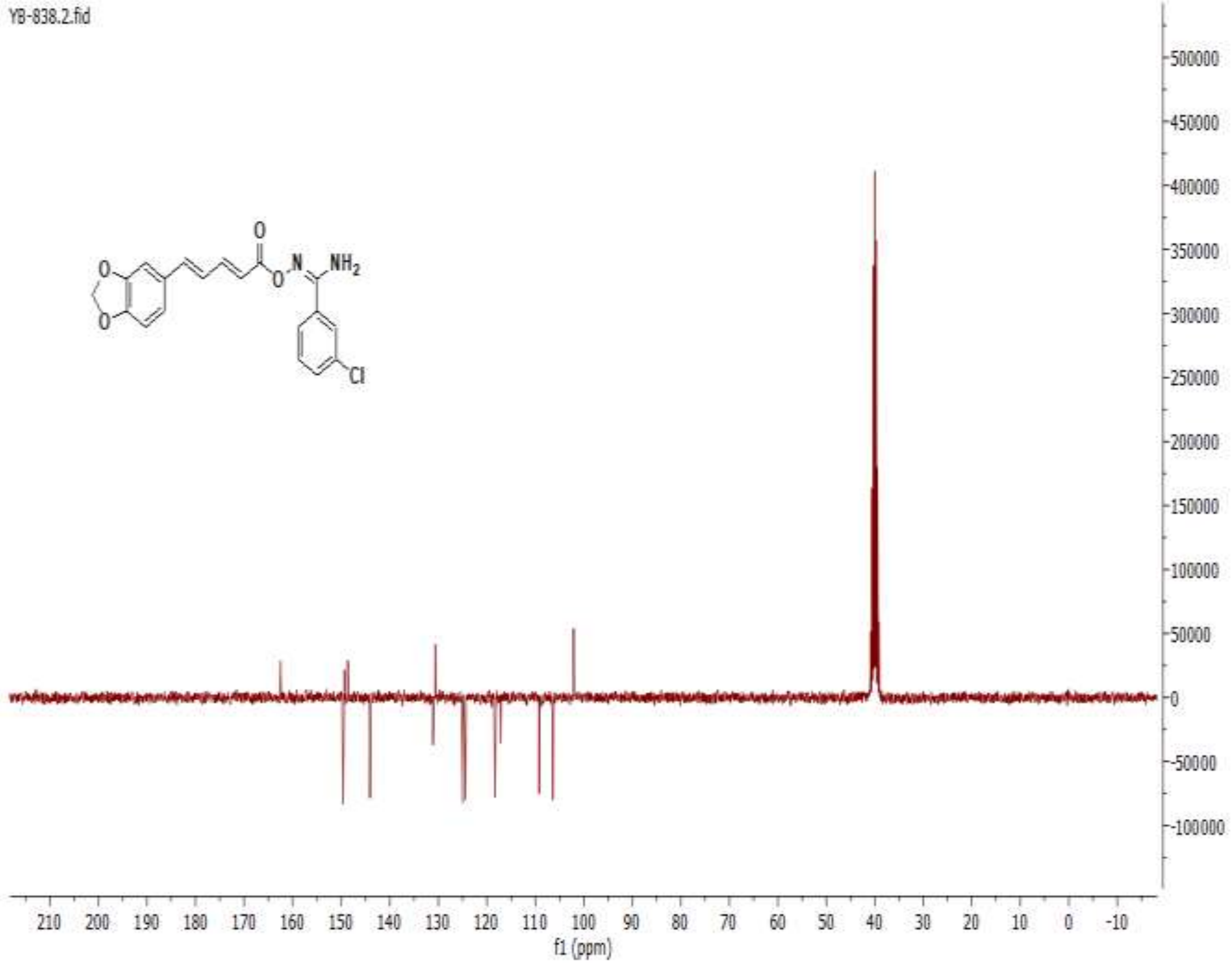

^{13}C NMR (100 MHz, δ ppm DMSO-d_6): 162.33, 149.95, 149.35, 144.26, 130.96, 130.66, 124.96, 124.36, 118.28, 117.06, 109.70, 106.28, 101.87

Information Classification: General

IR spectrum of (VIh) :

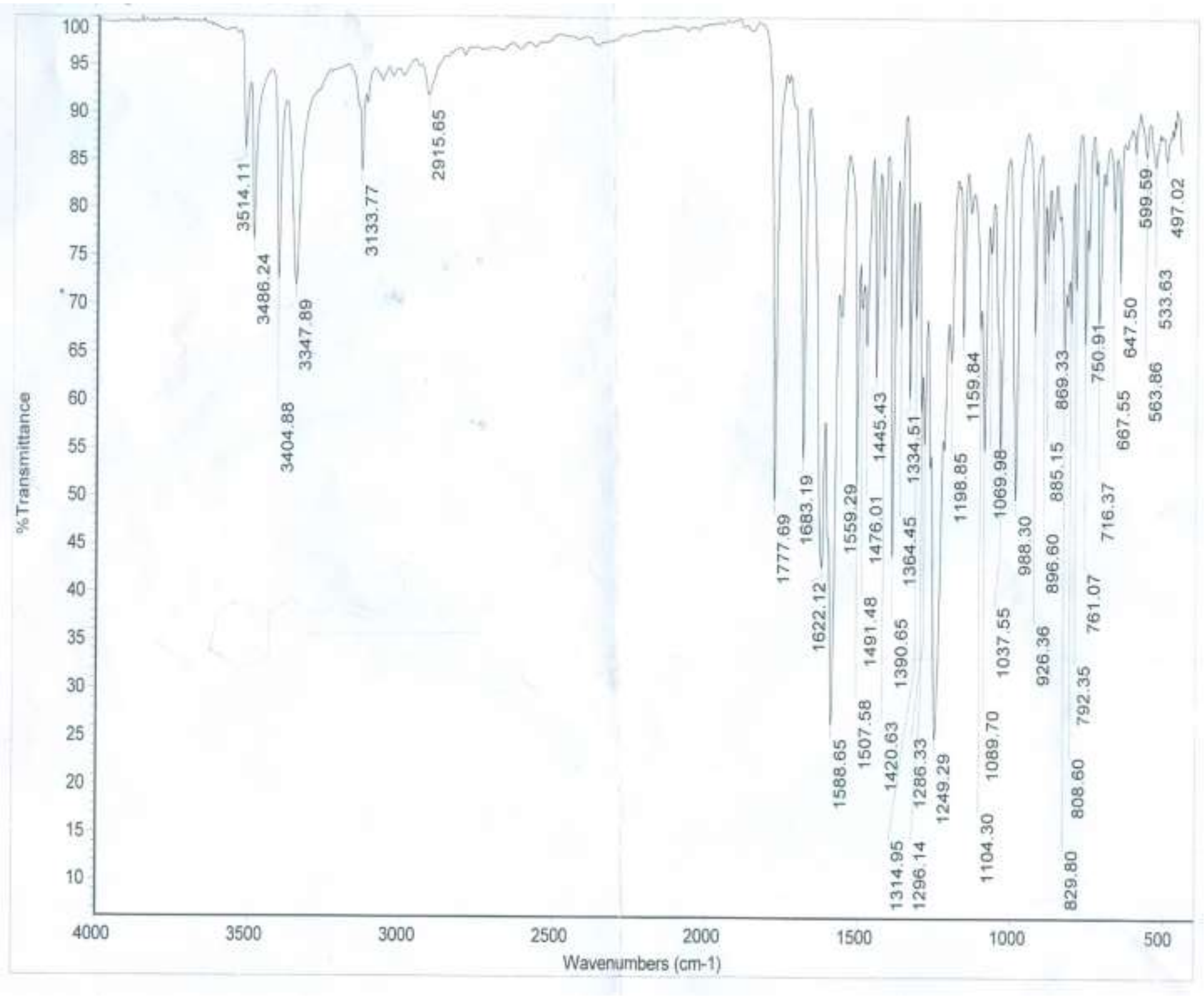

Information Classification: General

^{1}H NMR spectrum of (VIh) :

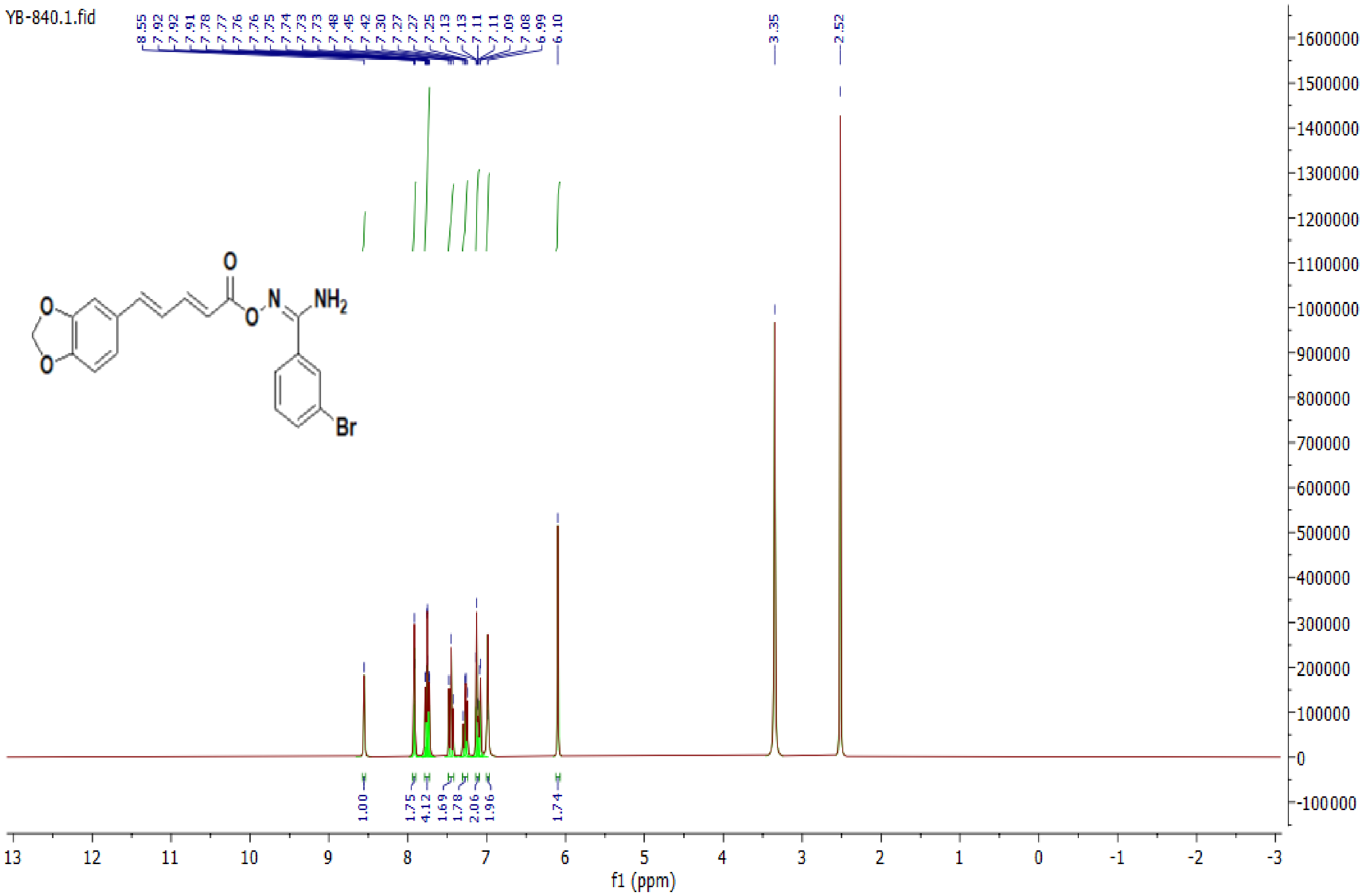

^{1}H NMR (400 MHz, δ ppm DMSO-d_6): 8.55 (s, 1H, Ar-H), 7.92 (s, 2H, Ar-H), 7.75 (t, J= 6.74 Hz, 4H, Ar-H), 7.45 (t, J= 7.9 Hz, 1H, Ar-H), 7.45 (t, J= 7.9 Hz, 1H, CH=CH), 7.27 (t, J= 7.7 Hz, 1H, Ar-H), 7.27 (t, J= 7.7 Hz, 1H, CH=CH), 7.13-7.07 (m, 2H, CH=CH), 6.98 (s, broad, 2H, NH2), 6.09 (s, 2H, O-CH_2-O)

Information Classification: General

^{13}C NMR spectrum of (VIh) :

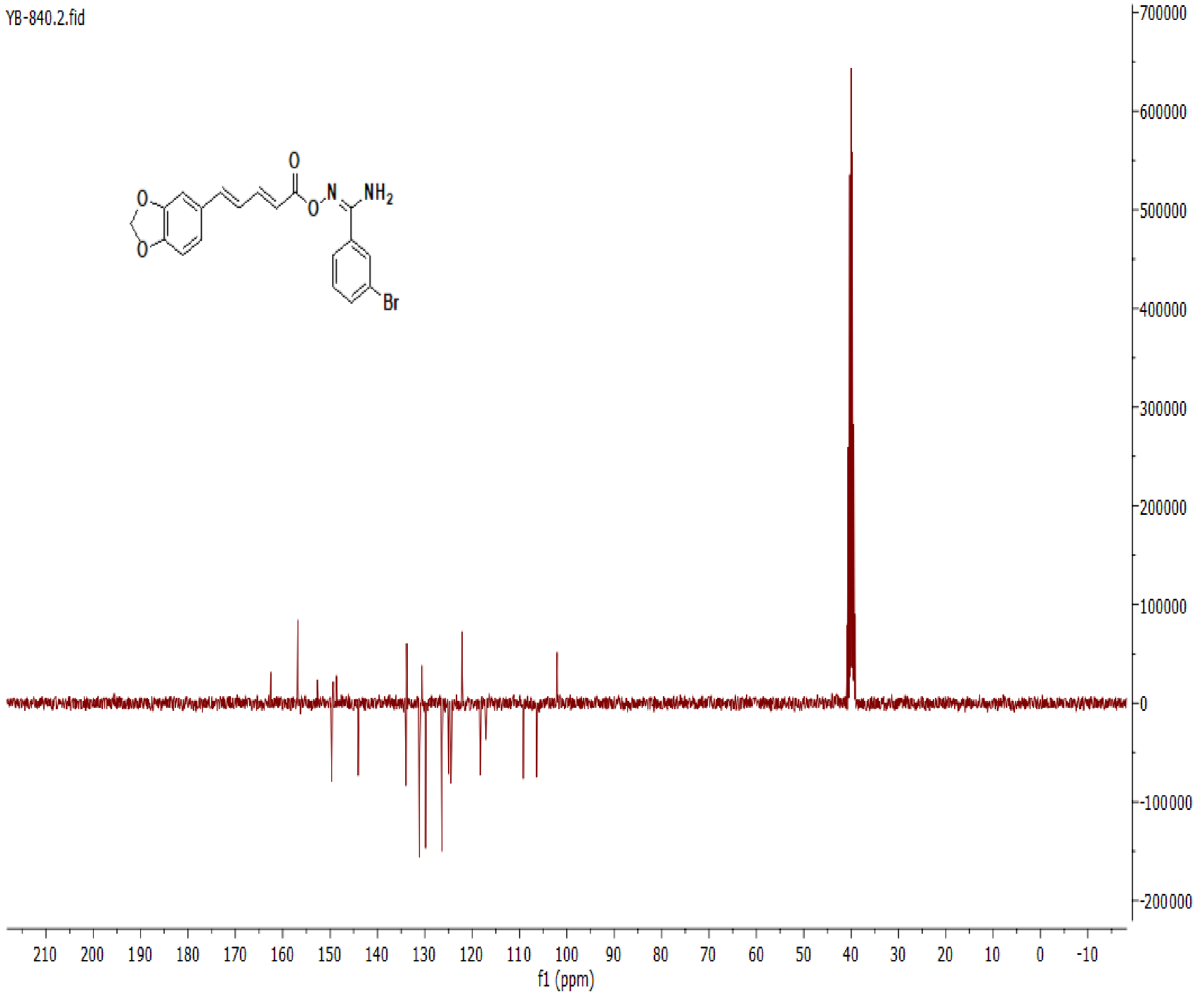

^{13}C NMR (100 MHz, δ ppm DMSO-d_6): 162.33, 156.64, 152.46, 149.65, 148.36, 143.95, 133.85, 131.27, 130.36, 129.67, 126.25, 124.96, 124.36, 122.08, 118.28, 117.37, 109.17, 106.28, 101.87

IR spectrum of (VIi) :

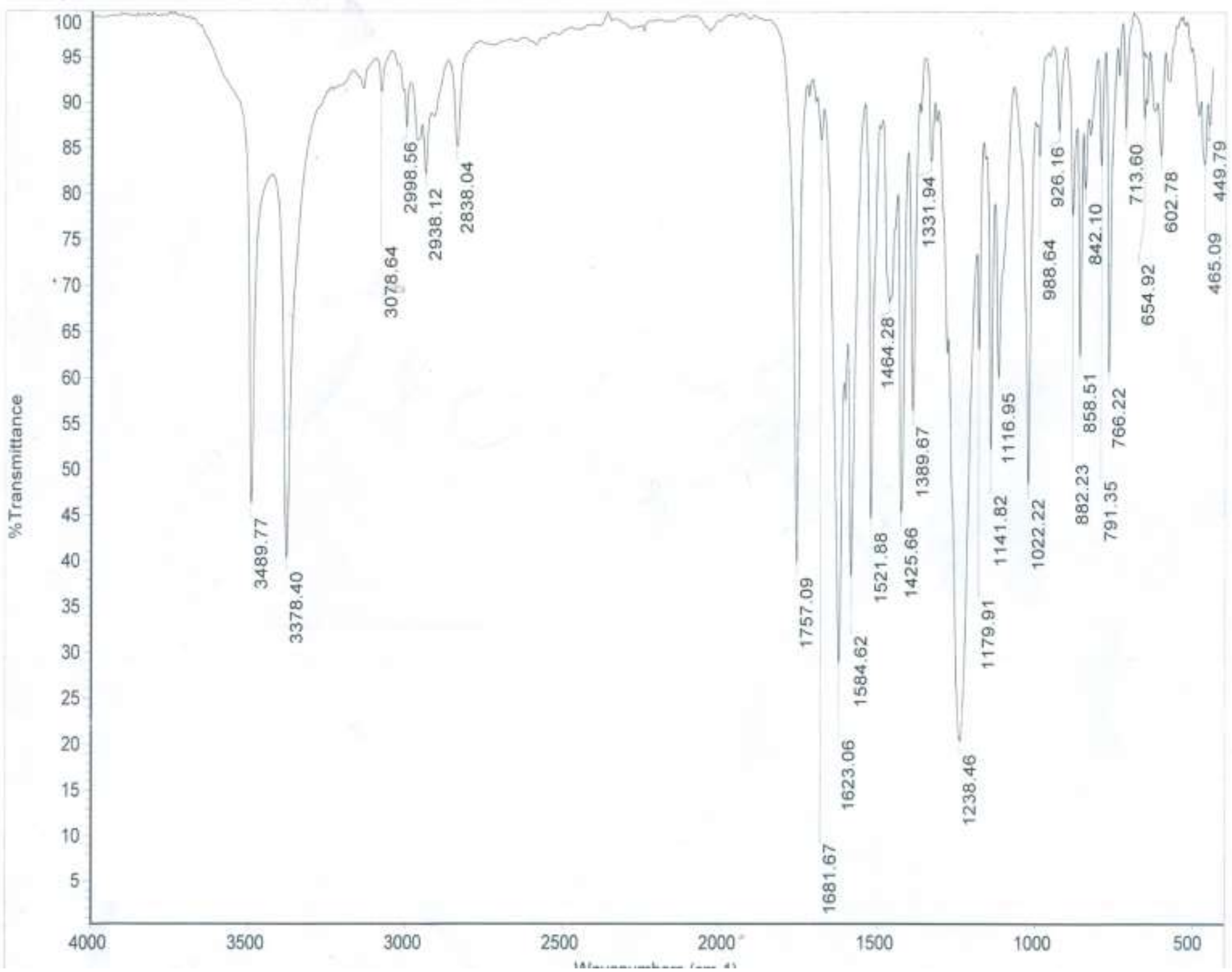

^{1}H NMR spectrum of (VIi) :

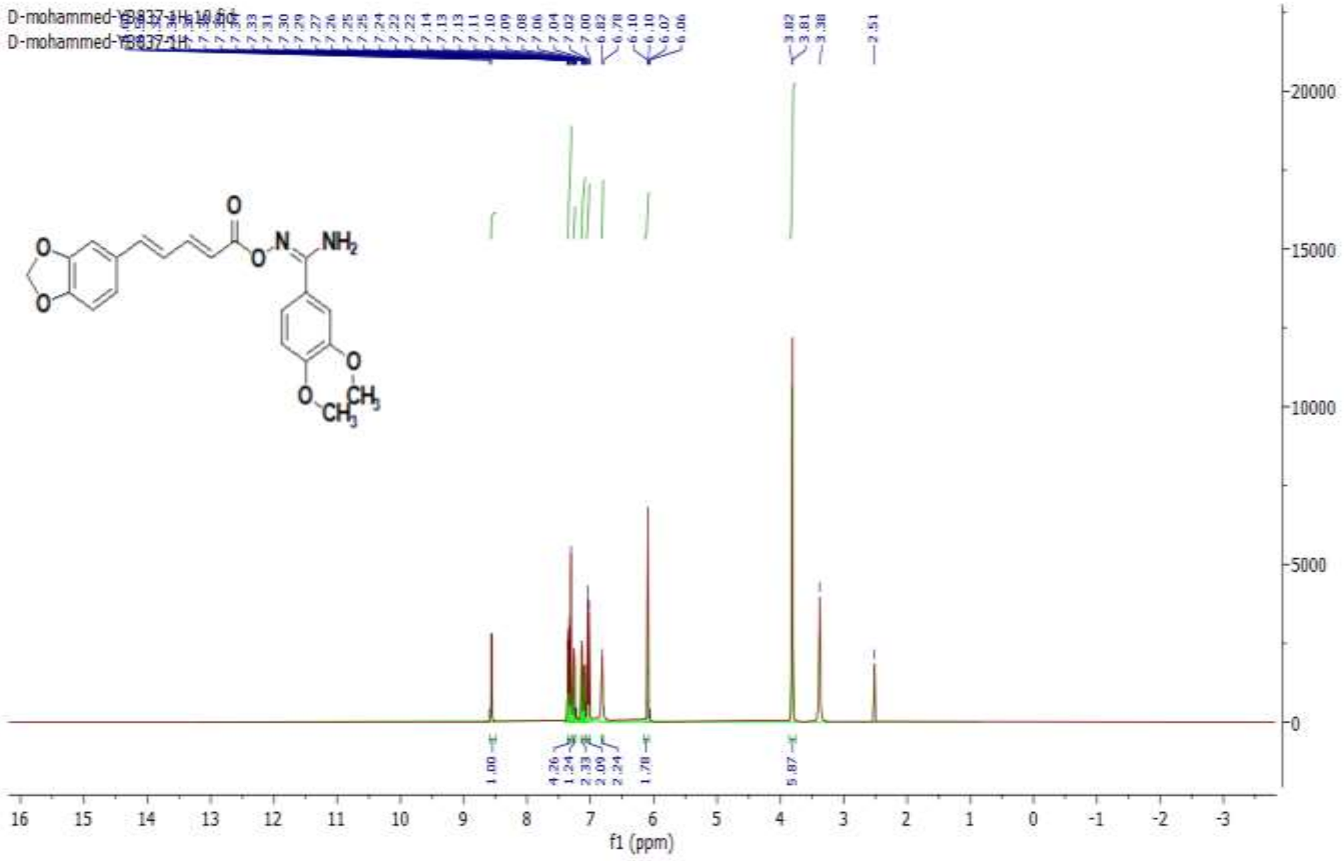

^{1}H NMR (400 MHz, δ ppm DMSO-d_6): 8.56 (s, 1H, Ar-H), 7.36-7.29 (m, 4H, Ar-H), 7.25 (d, J= 7.33 Hz, 1H, Ar-H), 7.15-7.08(m, 2H, CH=CH), 7.03 (d, J= 8.34 Hz, 2H, CH=CH), 6.81 (s, broad, 2H, NH2), 6.09 (s, 2H, O-CH_2-O), 3.81 (s, 6H, -$(OCH_3)_2$)

^{13}C NMR spectrum of (VIi) :

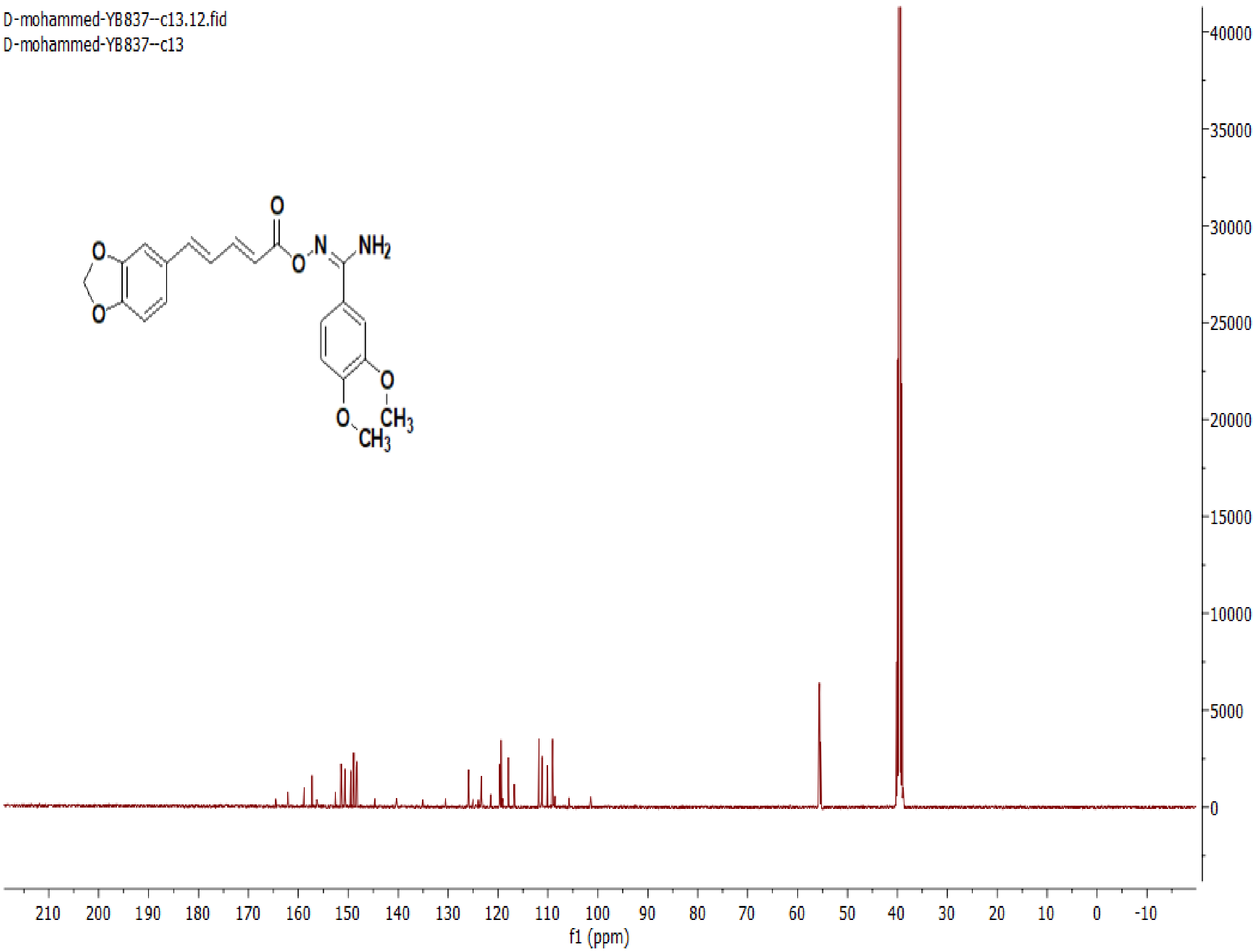

^{13}C NMR (100 MHz, δ ppm DMSO-d_6): 162.14, 158.69, 157.38, 152.55, 151.32, 150.63, 149.40, 148.71, 148.10, 125.69, 123.16, 119.63, 117.71, 116.79, 111.95, 111.03, 110.04, 109.12, 55.70

Information Classification: General

^{1}H NMR spectrum of (VIj) :

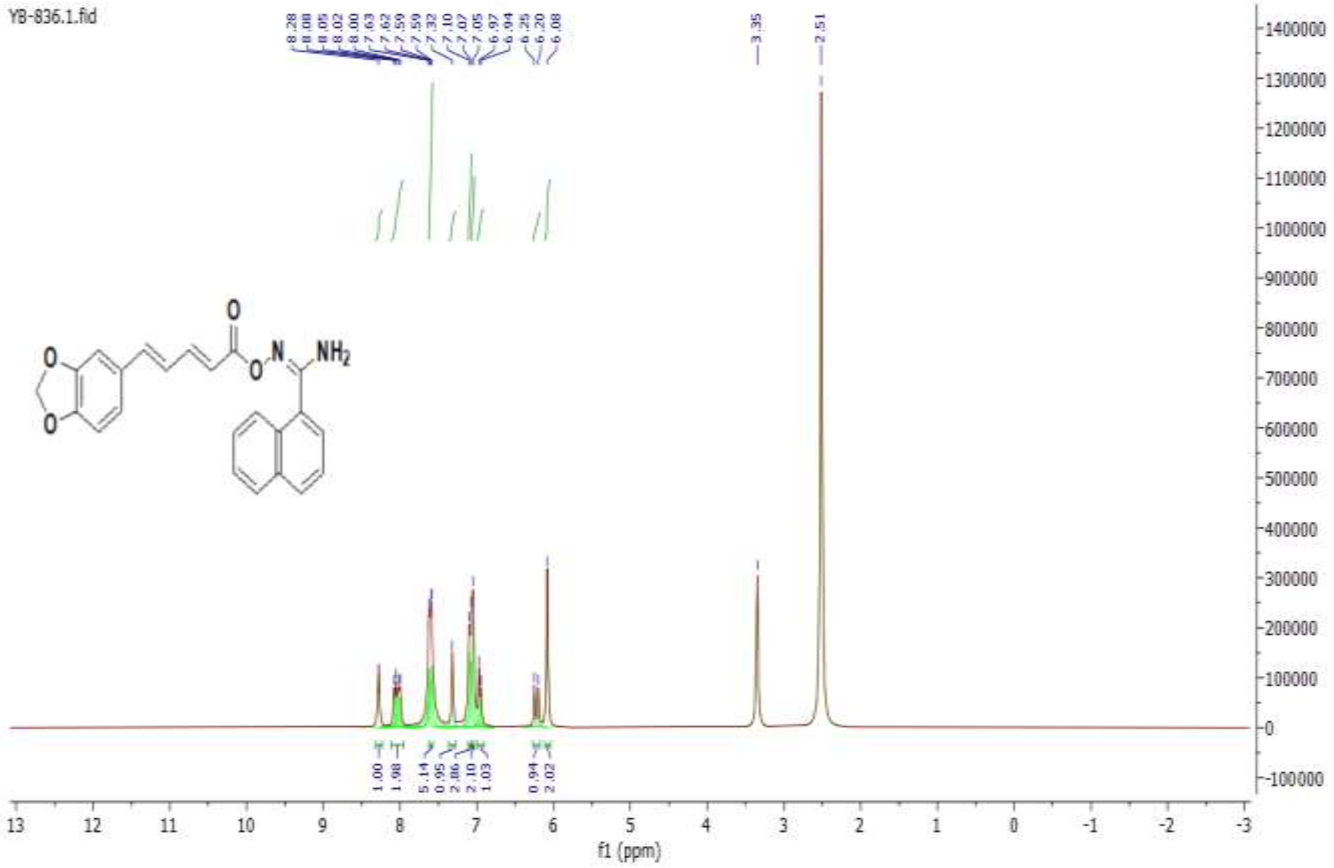

^{1}H NMR (400 MHz, δ ppm DMSO-d_6): 8.56 (s, 1H, Ar-H), 8.00 (m, 2H, Ar-H), 7.59 (m, 5H, Ar-H), 7.31 (s, 1H, Ar-H), 7.07 (m, 3H, CH=CH), 7.05 (s, broad, 2H, NH2), 6.95 (d, J= 8.0 Hz, 1H, Ar-H), 6.22 (d, J= 15.3 Hz, 1H, CH=CH), 6.08 (s, 2H, O-CH_2-O)

Information Classification: General

^{13}C NMR spectrum of (VIj) :

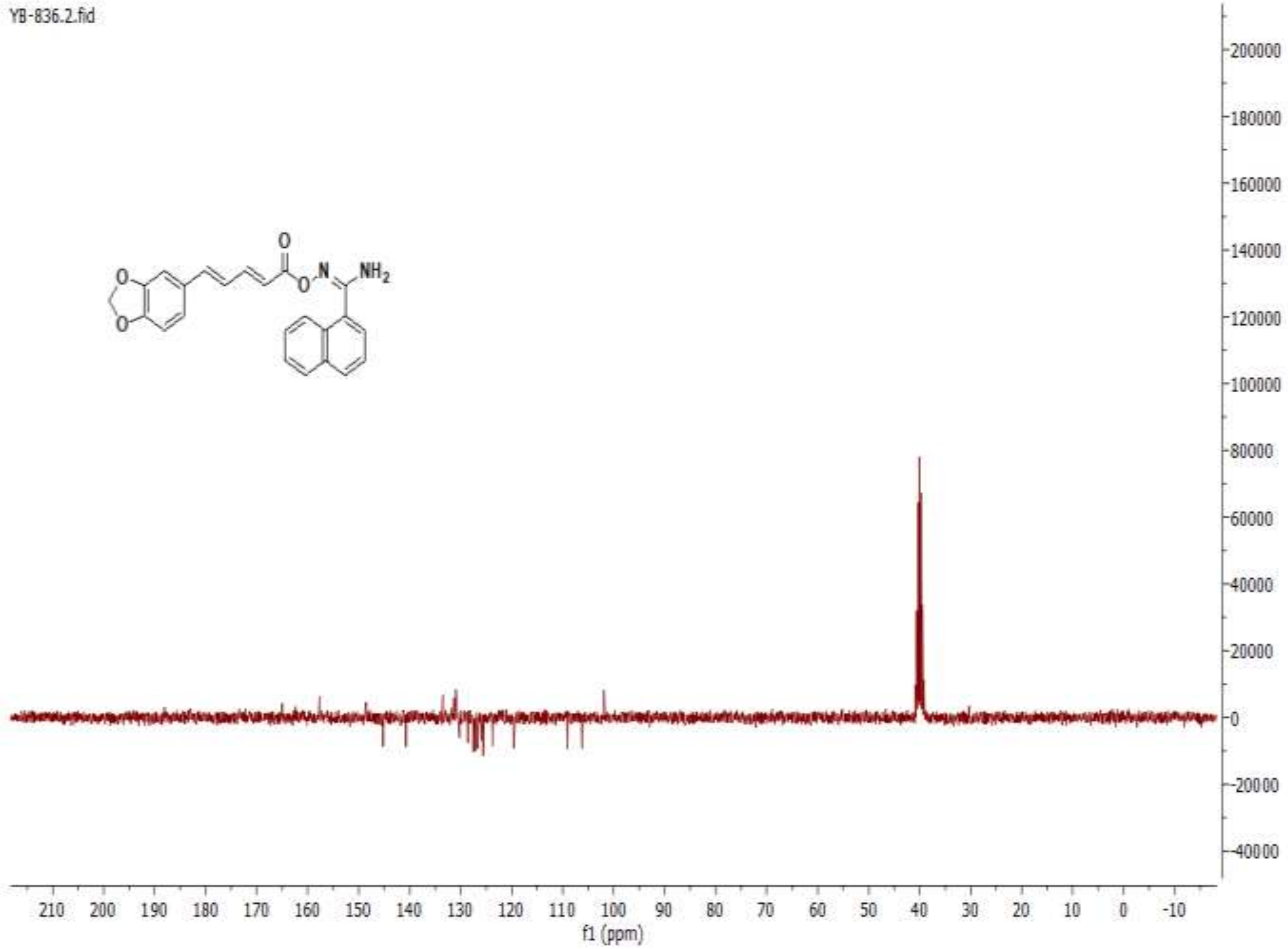

^{13}C NMR (100 MHz, δ ppm DMSO-d_6):164.84, 157.55, 148.66, 145.25, 140.76, 133.47, 131.27, 130.05, 128.46, 127.47, 127.17, 126.56, 126.25, 125.57, 123.98, 119.57, 109.02, 106.25, 101.87

Information Classification: General

IR spectrum of (VIk):

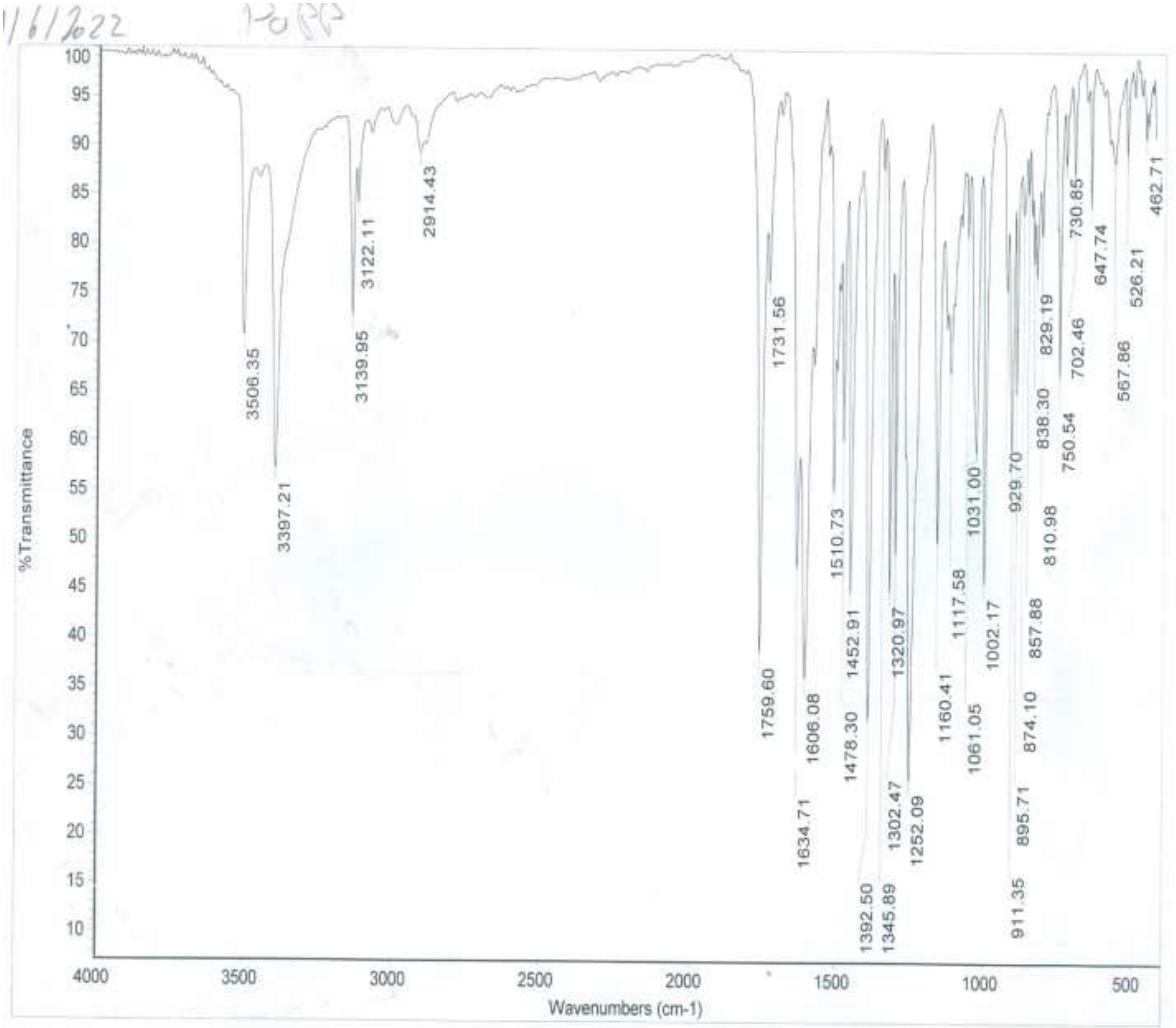

^{1}H NMR spectrum of (VIk) :

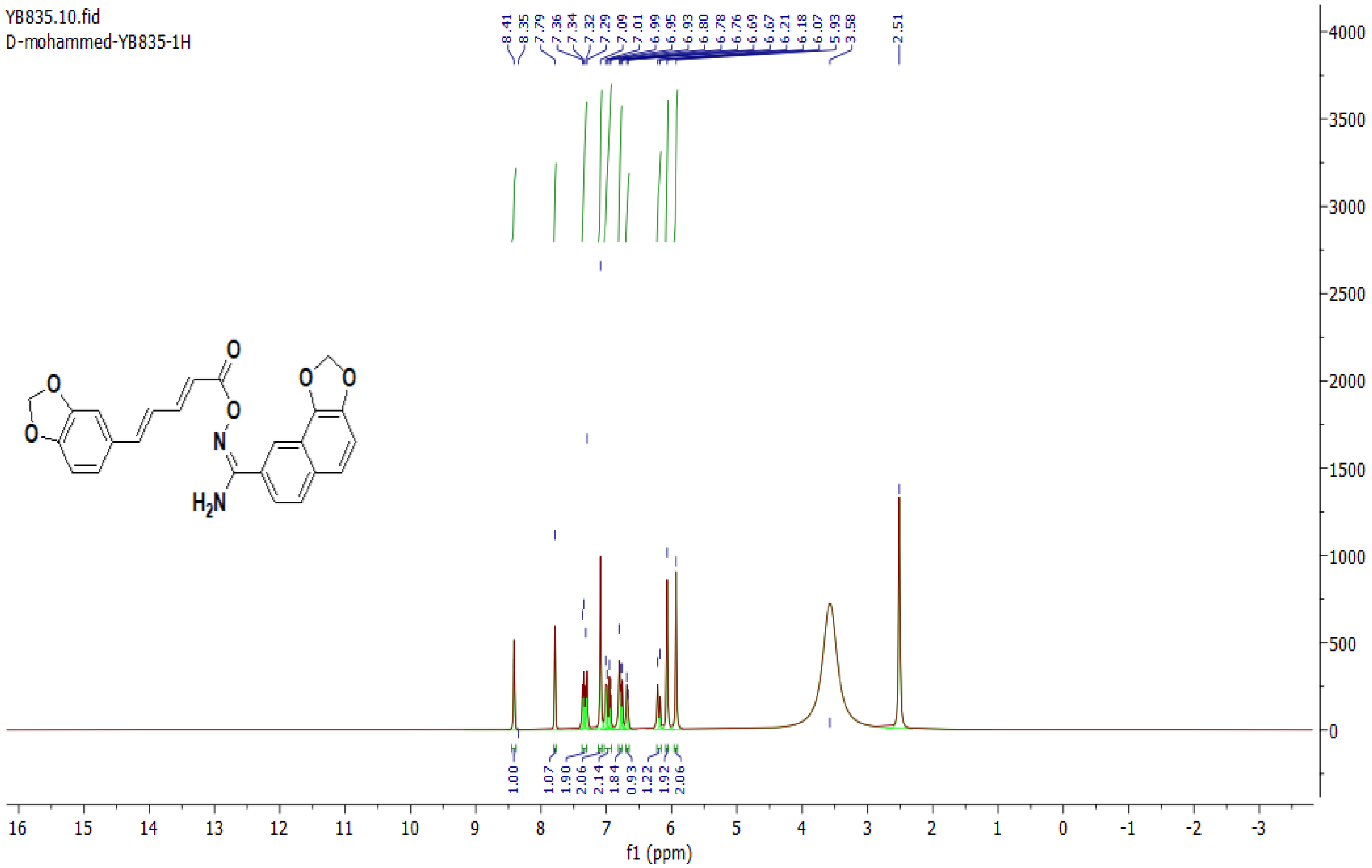

^{1}H NMR (400 MHz, δ ppm DMSO-d_6): 8.41 (s, 1H, Ar-H), 7.79 (s, 1H, Ar-H), 7.4-7.3 (m, 2H, Ar-H), 7.00 (s, broad, 2H, NH2), 6.95(dd, J = 8.3 Hz , 7.7 Hz, 2H, Ar-H), 6.74-6.82 (m, 2H, CH=CH), 6.68 (d, J = 8 Hz, 1H, CH=CH), 6.19 (d, J = 15.4 Hz, 1H, CH=CH), 6.06 (s, 2H, O-CH_2-O), 5.93 (s, 2H, O-CH_2-O)

Information Classification: General

^{13}C NMR spectrum of (VIj) :

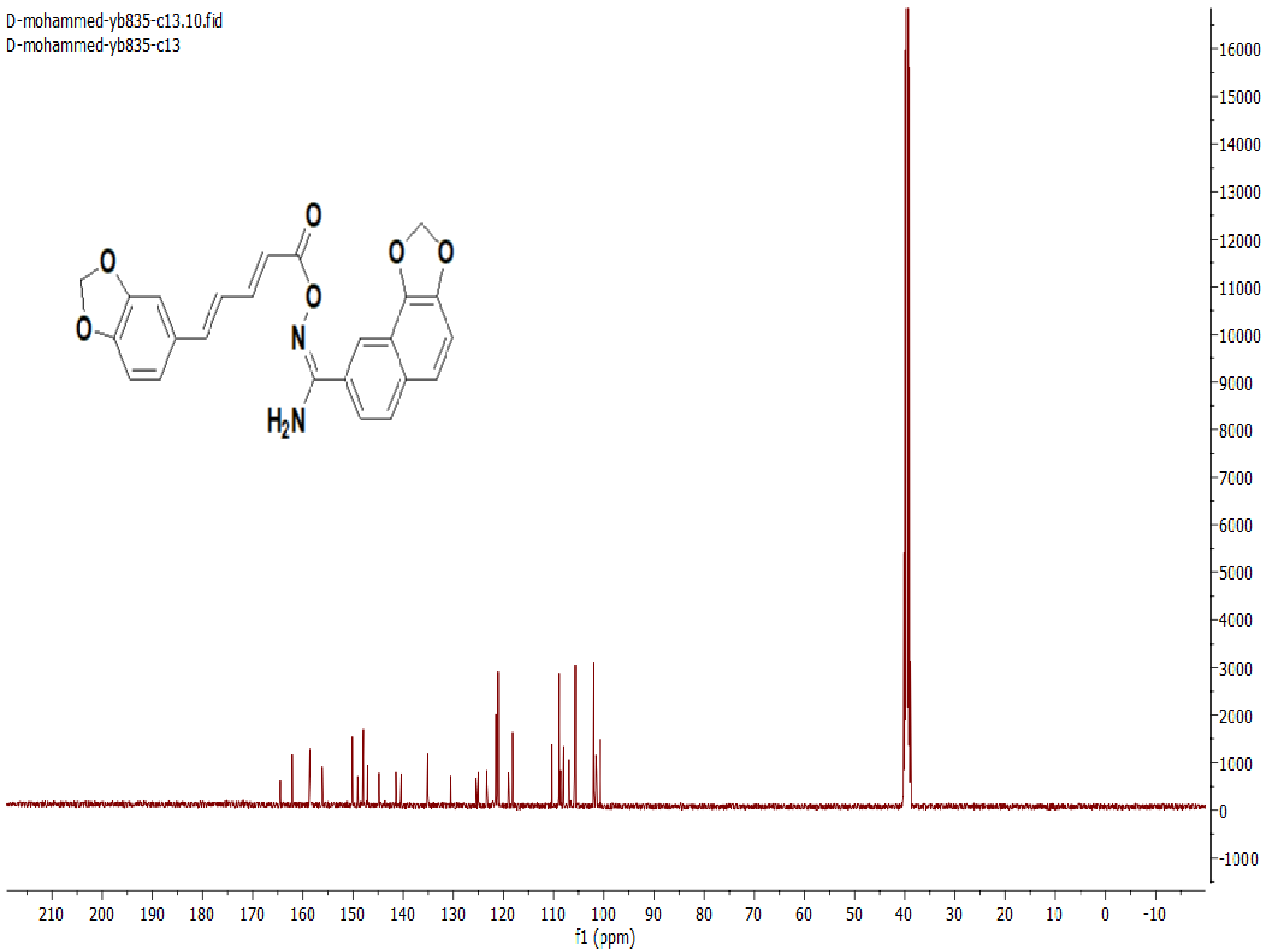

^{13}C NMR (100 MHz, δ ppm DMSO-d_6): 164.46, 162.16, 158.71, 156.40, 150.04, 149.12, 147.81, 147.20, 144.59, 141.44, 140.44, 135.00, 130.54, 125.40, 125.01, 123.18, 121.57, 120.95, 119.03, 118.04, 110.36, 108.83, 108.14, 107.83, 105.60, 102.07, 101.58, 100.77

Information Classification: General

Appendix A

Experimental procedures

General Details

All the chemicals used were of analytical grade and purified by standard methods prior to use. Silica gel column chromatography was carried out using kieselgel 60 (Merck). TLC analysis was performed on aluminium-backed plates coated with silica gel 60 F_{254} (Merck). Melting points were determined using a Gallen Kamp melting point apparatus and are uncorrected. Components were visualized using potassium permanganate solution and UV light. NMR Spectra were taken using a Varian Unity INOVA 400 MHz and Bruker AC250 MHz spectrometers for proton and carbon at university of Aberdeen. All numbers referring to NMR data obtained are in parts per million (ppm).

4.2. Biological evaluation

4.2.1. Cytotoxic activity using MTT Assay and evaluation of IC_{50}

4.2.1.1. MTT assay

MTT assay was carried out to study the effect of compounds on mammary epithelial cells (MCF-10A)[26,27]. The medium in which cells were propagated contained Dulbecco's modified Eagle's medium (DMEM)/ Ham's F-12 medium (1:1) supplemented with epidermal growth factor (20 ng/mL), hydrocortisone (500 ng/mL), insulin (10 µg/mL), 2 mM glutamine and 10% foetal calf serum. After every 2-3 days, the cells were passaged using trypsin ethylenediamine tetra acetic acid (EDTA). The cells were seeded at a density of 10^4 cells mL^{-1} in flat-bottomed culture plates containing 96 wells each. After 24 h, medium was removed from the plates and the compounds in (in 0.1% DMSO) were

Information Classification: General

added (in 200 µL medium to yield a final concentration of 0.1% v/v) to the wells of plates. A single compound was designated with four wells followed by incubation of plates for 96h at 37°C. After incubation, medium was removed completely from the plates followed by addition of MTT (0.4 mg/mL in medium) to each well and subsequent incubation of plates for 3h. MTT (along with the medium) was removed and DMSO (150µL) was added to each well of the culture plates, followed by vortexing and subsequent measurement of absorbance (at 540 nm) using microplate reader. The data are shown as percentage inhibition of proliferation in comparison with controls containing 0.1% DMSO.

4.2.1.2. Assay for antiproliferative effect

To explore the antiproliferative potential of compounds MTT assay was performed according to previously reported procedure[28,29] using different cell lines to explore the antiproliferative potential of compounds propidium iodide fluorescence assay was performed using different cell lines. To calculate the total nuclear DNA, a fluorescent dye (propidium iodide, PI) is used which can attach to the DNA, thus offering a quick and precise technique. PI cannot pass through the cell membrane and its signal intensity can be considered as directly proportional to quantity of cellular DNA. Cells whose cell membranes are damaged or have changed permeability are counted as dead ones. The assay was performed by seeding the cells of different cell lines at a density of 3000-7500 cells/well (in 200µl medium) in culture plates followed by incubation for 24h at 37 °C in humidified 5% CO_2/95% air atmospheric conditions. The medium was removed; the compounds were added to the plates at 10 µM concentrations (in 0.1% DMSO) in triplicates, followed by incubation for 48 h. DMSO (0.1%) was used as control. After

Information Classification: General

incubation, medium was removed followed by the addition of PI (25 µl, 50µg/mL in water/medium) to each well of the plates. At -80 °C, the plates were allowed to freeze for 24 h, followed by thawing at 25°C. A fluorometer (Polar-Star BMG Tech) was used to record the readings at excitation and emission wavelengths of 530 and 620 nm for each well. The percentage cytotoxicity of compounds was calculated using the following formula:

$$\% \text{ Cytotoxicity} = \frac{A_C - A_{TC}}{A_C} \times 100$$

Where A_{TC}= Absorbance of treated cells and A_C= Absorbance of control. Erlotinib was used as positive control in the assay.

4.2.1.3. EGFR inhibitory assay

EGFR-TK assay was performed to evaluate the inhibitory potency of the tested compounds against EGFR[30]. Baculoviral expression vectors including pBlueBacHis2B and pFASTBacHTc were used separately to clone 1.6 kb cDNA coding for EGFR cytoplasmic domain (EGFR-CD, amino acids 645–1186). 5′ upstream to the EGFR sequence comprised a sequence that encoded $(His)_6$. Sf-9 cells were infected for 72h for protein expression. The pellets of Sf-9 cells were solubilized in a buffer containing sodium vanadate (100 µM), aprotinin (10 µg/mL), triton (1%), HEPES buffer (50mM), ammonium molybdate (10 µM), benzamidine HCl (16 µg/mL), NaCl (10 mM), leupeptin (10 µg/mL) and pepstatin (10 µg/mL) at 0°C for 20 min at pH 7.4, followed by centrifugation for 20 min. To eliminate the non-specifically bound material, a Ni-NTA super flow packed column was used to pass through and wash the crude extract supernatant first with 10 mM and then with 100 mM imidazole. Histidine-linked proteins

Information Classification: General

were first eluted with 250 and then with 500 mM imidazole subsequent to dialysis against NaCl (50 mM), HEPES (20 mM), glycerol (10%) and 1 µg/mL each of aprotinin, leupeptin and pepstatin for 120 min. The purification was performed either at 4 °C or on ice. To record autophosphorylation level, EGFR kinase assay was carried out on the basis of DELFIA/Time-Resolved Fluorometry. The compounds were first dissolved in DMSO absolute, subsequent to dilution to appropriate concentration using HEPES (25 mM) at pH 7.4. Each compound (10 µL) was incubated with recombinant enzyme (10 µL, 5 ng for EGFR, 1:80 dilution in 100 mM HEPES) for 10 min at 25°C, subsequent to the addition of 5X buffer (10 µL, containing 2 mM $MnCl_2$, 100 µM Na_3VO_4, 20 mM HEPES and 1 mM DTT) and ATP-$MgCl_2$ (20 µL, containing 0.1 mM ATP and 50 mM $MgCl_2$) and incubation for 1h. The negative and positive controls were included in each plate by the incubation of enzyme either with or without ATP-$MgCl_2$. The liquid was removed after incubation and the plates were washed thrice using wash buffer. Europium-tagged antiphosphotyrosine antibody (75 µL, 400 ng) was added to each well followed by incubation of 1h and then washing of the plates using buffer. The enhancement solution was added to each well and the signal was recorded at excitation and emission wavelengths of 340 at 615 nm. The autophosphorylation percentage inhibition by compounds was calculated using the following equation:

$$100\% - [(negative\ control)/(positive\ control) - (negative\ control)]$$

Using the curves of percentage inhibition of eight concentrations of each compound, IC_{50} was calculated. Majority of signals detected by antiphosphotyrosine antibody were from EGFR because the enzyme preparation contained low impurities.

Information Classification: General

4.2.1.4. BRAF kinase assay

V^{600E} mutant BRAF kinase assay was performed to investigate the activity of the most active compounds against BRAF[31]. Mouse full-length GST-tagged $BRAF^{V600E}$ (7.5 ng, Invitrogen, PV3849) was pre-incubated with drug (1 µL) and assay dilution buffer (4 µL) for 60 min at 25°C. In assay dilution buffer, a solution (5 µL) containing $MgCl_2$ (30 mM), ATP (200 µM), recombinant human full length (200 ng) and *N*-terminal His-tagged MEK1 (Invitrogen) was added to start the assay, subsequent to incubation for 25 min at 25°C. The assay was stopped using 5X protein denaturing buffer (LDS) solution (5 µL). To further denature the protein, heat (70° C) was applied for 5 min. 4-12% precast NuPage gel plates (Invitrogen) were used to carry out electrophoresis (at 200 V). 10 µL of each reaction was loaded into the precast plates and electrophoresis was allowed to proceed. After completion of electrophoresis, the front part of the precast gel plate (holding hot ATP) was cut and afterwards cast-off. The dried gel was developed using a phosphor screen. A reaction without active enzyme was used as negative control while that containing no inhibitor served as positive control. To study the effect of compounds on cell-based pERK1/2 activity in cancer cells, commercially available ELISA kits (Invitrogen) were used according to manufacturer's instructions.

4.2.1.5. CDK2 inhibitory assay

Protein Kinase Assays. Human CDK2/A2 was purchased from New England Biolabs IC_{50} values for CDK2/A were determined according to the supplier's instructions (Upstate). CDK2/A were assayed at an ATP concentration of 12.5 µM.[32]

Information Classification: General

4.3. Statistical analysis

Computerized Prism 5 program was used to statistically analyzed data using one-way ANOVA test followed by Tukey's as post ANOVA for multiple comparison at $P \leq .05$. Data were presented as mean ± SEM.

Information Classification: General

Fig. 2

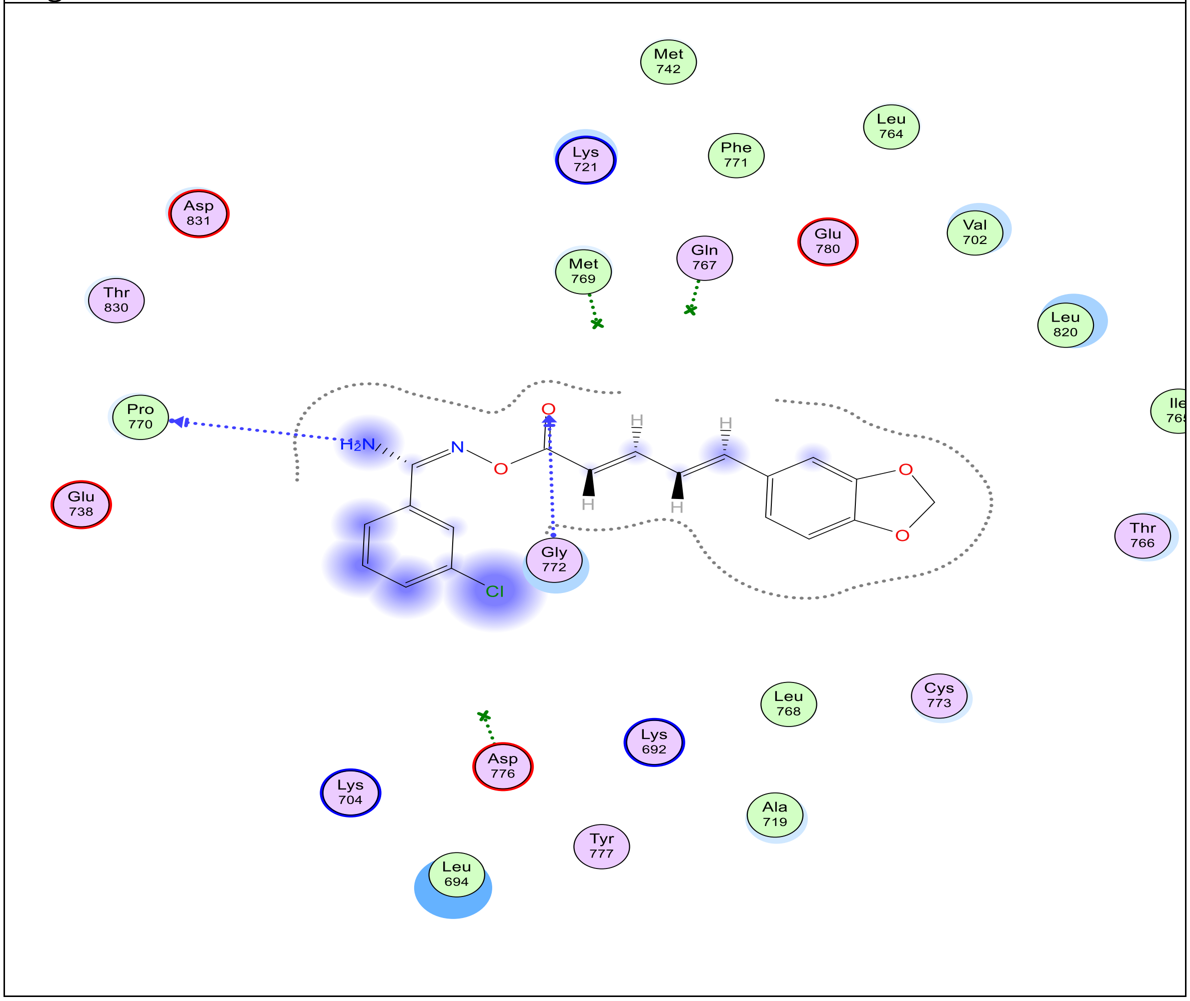

Information Classification: General

Fig. 3a

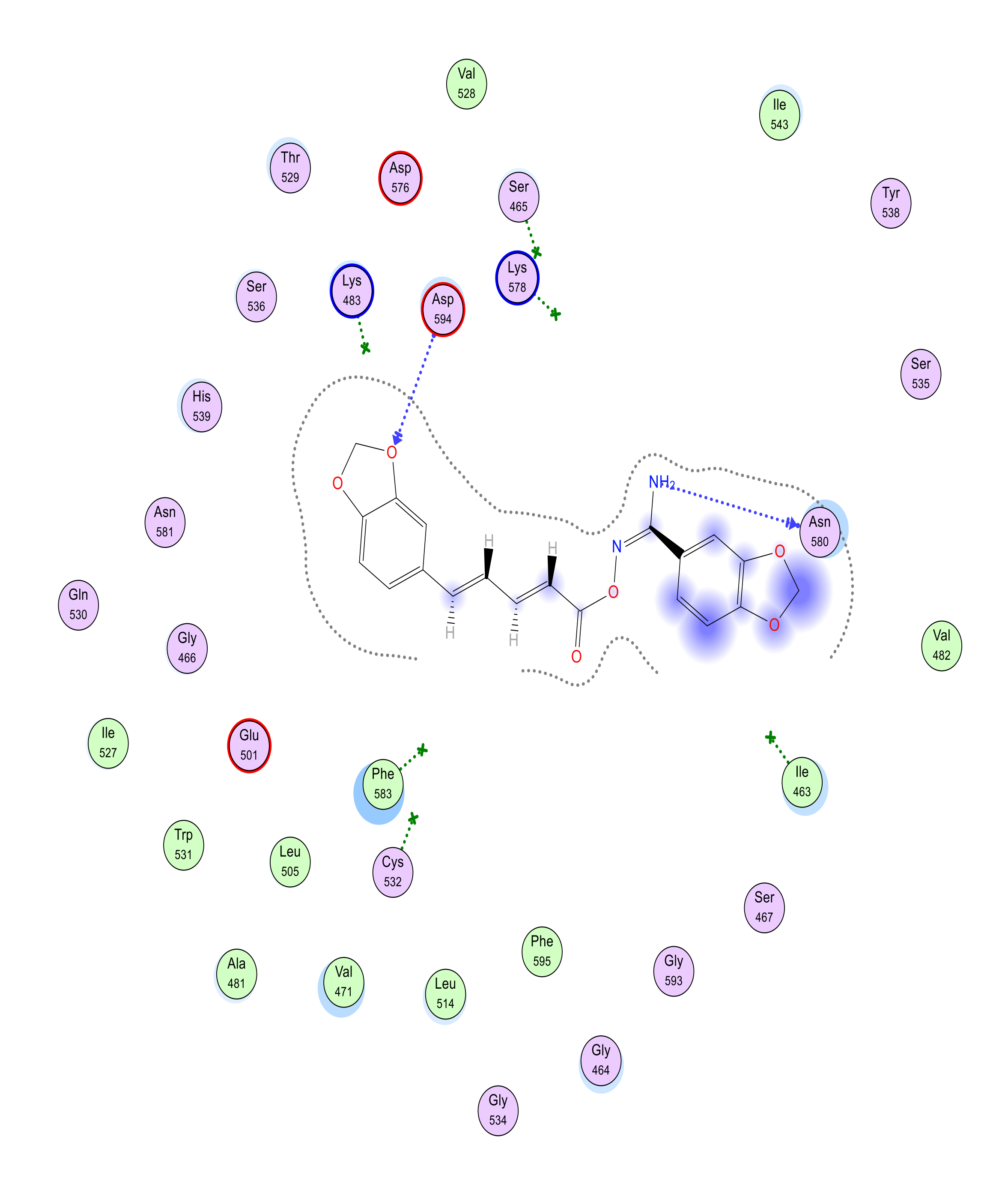
Val 528
Ile 543
Thr 529
Asp 576
Ser 465
Tyr 538
Ser 536
Lys 483
Asp 594
Lys 578
Ser 535
His 539
Asn 581
Asn 580
Gln 530
Gly 466
Val 482
Ile 527
Glu 501
Phe 583
Ile 463
Trp 531
Leu 505
Cys 532
Ser 467
Phe 595
Ala 481
Val 471
Leu 514
Gly 593
Gly 464
Gly 534

Fig. 3b

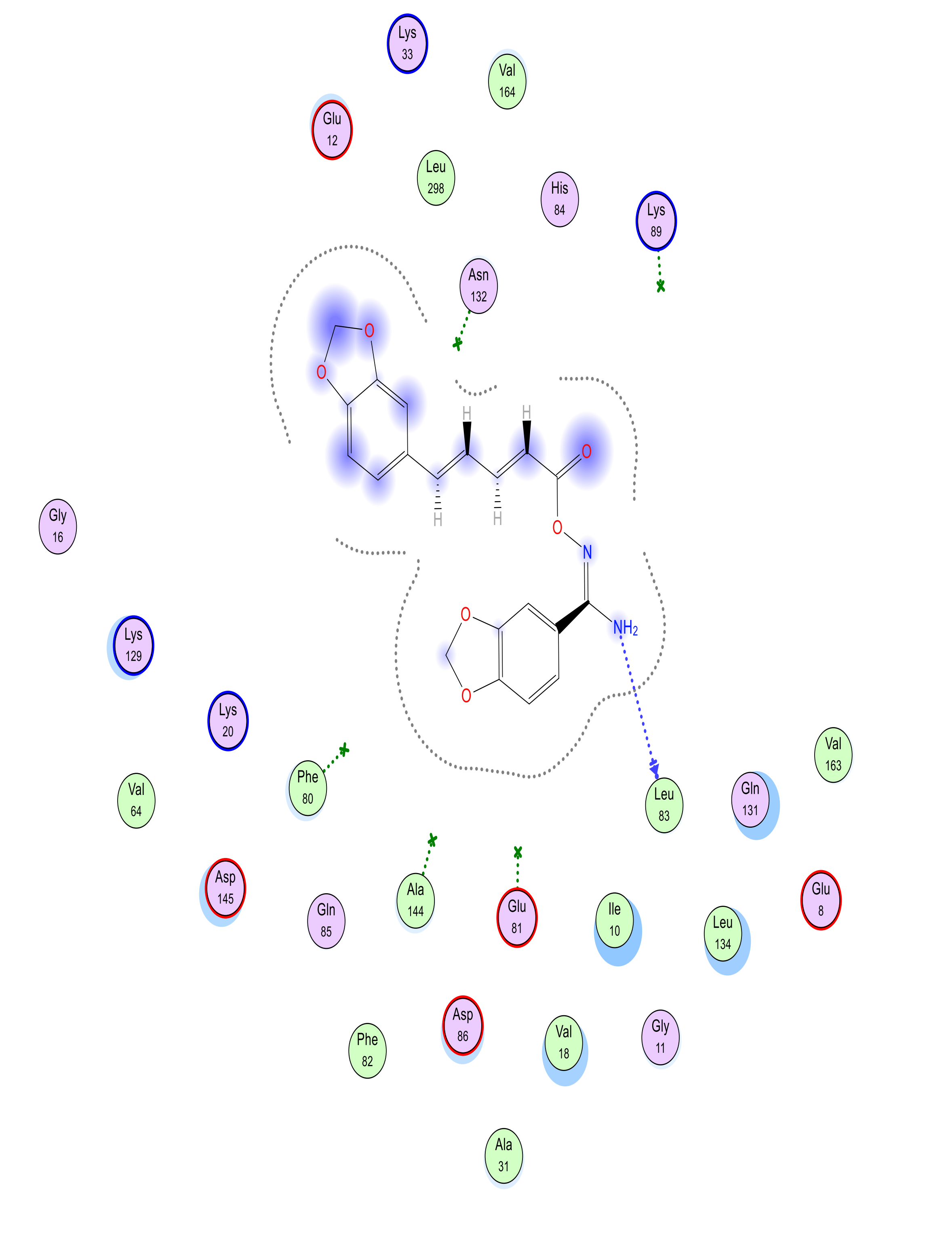
Lys 33
Val 164
Glu 12
Leu 298
His 84
Lys 89
Asn 132
O
O
H
H
O
H
H
O
N
O
O
NH2
Gly 16
Lys 129
Lys 20
Phe 80
Leu 83
Gln 131
Val 163
Val 64
Asp 145
Gln 85
Ala 144
Glu 81
Ile 10
Leu 134
Glu 8
Phe 82
Asp 86
Val 18
Gly 11
Ala 31

Molecular Docking Simulation Data

Table S1. Molecular docking of **VIc**, **VIf**, **VIg**, **VIi**, &**VIk** within active sites of EGFR (PDB ID: 1M17), BRAF^{V600E} (PDB ID: 4MNF), and CDK2 (PDB ID: 1PYE); respectively.

Cpd	EGFR (PDB ID: 1M17)					BRAF^{V600E} (PDB ID: 4MNF)					CDK2 (PDB ID: 1PYE)				
	S^{a}	RMSD (Å)	Binding Interactions a.a. residue	Type	Distance (Å)	S	RMSD (Å)	Binding Interactions a.a. residue	Type	Distance (Å)	S	RMSD (Å)	Binding Interactions a.a. residue	Type	Distance (Å)
**VIc**	-6.02	1.52	PRO 770	H-donor	3.21	-5.61	1.40	SER 465	H-donor	2.56	-5.58	1.51	ASN 132	H-donor	2.72
													LYS 89	H-acceptor	3.03
**VIf**	-6.53	1.97	MET 742	H-donor	3.23	-6.59	1.83	ASN 580	H-donor	3.04	-5.72	1.78	GLU 81	H-donor	3.17
			LYS 721	pi-H	3.74			ASN 580	H-donor	2.83			ALA 144	pi-H	4.47
			GLY 772	pi-H	3.60			ASP 594	H-acceptor	2.91					
**VIg**	-6.72	1.15	PRO 770	H-donor	3.06	-6.70	1.63	ASN 580	H-donor	2.97	-5.45	1.70	LEU 83	H-donor	2.91
			GLY 772	H-acceptor	3.25			ASP 594	H-acceptor	2.91					
**VIi**	-5.84	1.96	GLU 738	H-donor	2.89	-6.24	2.00	CYS 532	H-acceptor	2.88	-5.54	1.73	LEU 83	H-donor	3.17
			LYS 721	H-acceptor	3.08			VAL 471	pi-H	3.06			LYS 89	H-acceptor	3.14
			LYS 721	H-acceptor	2.88										
**VIk**	-6.22	2.82	GLY 772	pi-H	4.27	-6.67	2.06	ASN 580	H-donor	2.98	-5.77	2.06	LEU 83	H-donor	2.88

Information Classification: General

								ASN 580	H-donor	2.82					
								ASP 594	H-acceptor	2.91					
**Ref.**[b]	-7.30	1.28	GLN 767	H-donor	3.15	-7.72	0.63	CYS 532	H-acceptor	2.75	-5.89	1.84	GLU 81	H-donor	3.05
			MET 769	H-acceptor	2.70								LEU 83	H-acceptor	3.07

[a] S: docking score (Kcal/mol); [b] Ref: co-crystallized ligand Erlotinib for 1M17, **29L** [2-{4-[(1E)-1-(hydroxyimino)-2,3-dihydro-1H-inden-5-yl]-3-(38yridine-4-yl)-1H-pyrazol-1-yl}ethanol] for 4MNF, and Dinaciclib for 1PYE protein.

Information Classification: General

SwissADME Calculations

Table S2. ADME, Pharmacokinetic, and PAINS Computational Analysis of Compounds (VIa-k)

#	M.F.[a]	M.W.[b]	Nrotb[c]	HBA[d]	HBD[e]	MR[f]	TPSA[g]	iLogP[h]	Water solubility	HIA%[i]	BBB permeant[j]	Pgp substrate[k]	F[l]	PAINS[m]
**VIa**	$C_{19}H_{16}N_2O_4$	336	6	5	1	93.51	83.14	2.93	Moderately soluble	High	NO	NO	0.55	0
**VIb**	$C_{19}H_{15}ClN_2O_4$	371	6	5	1	98.52	83.14	3.25	Moderately soluble	High	NO	NO	0.55	0
**VIc**	$C_{19}H_{15}BrN_2O_4$	414	6	5	1	101.21	83.14	3.38	Moderately soluble	High	NO	NO	0.55	0
**VId**	$C_{20}H_{18}N_2O_5$	366	7	6	1	100.01	92.37	3.29	Moderately soluble	High	NO	NO	0.55	0
**VIe**	$C_{20}H_{18}N_2O_4$	350	6	5	1	98.48	83.14	3.24	Moderately soluble	High	NO	NO	0.55	0
**VIf**	$C_{19}H_{15}ClN_2O_4$	371	6	5	1	98.52	83.14	3.03	Moderately soluble	High	NO	NO	0.55	0
**VIj**	$C_{19}H_{15}ClN_2O_4$	371	6	5	1	98.52	83.14	3.28	Moderately soluble	High	NO	NO	0.55	0
**VIh**	$C_{19}H_{15}BrN_2O_4$	414	6	5	1	101.21	83.14	3.31	Moderately soluble	High	NO	NO	0.55	0
**VIi**	$C_{21}H_{20}N_2O_6$	396	8	7	1	106.5	101.6	3.41	Moderately soluble	High	NO	NO	0.55	0
**VIj**	$C_{22}H_{22}N_2O_7$	426	9	8	1	112.99	110.83	3.56	Moderately soluble	High	NO	NO	0.55	0
**VIk**	$C_{20}H_{16}N_2O_6$	380	6	7	1	99.58	101.6	3.13	Soluble	High	NO	NO	0.55	0
**RO5**		≤ 500	≤ 10	≤ 10	≤ 5	≤ 130	≤ 140	≤ 5						

[a] MF, Molecular Formula; [b] M.W., Molecular Wight; [c] nrotb, # of rotatable bonds; [d] HBA, Hydrogen Bond Acceptor; [e] HBD, Hydrogen bond donor; [f] MR, Molar Refractivity; [g] TPSA, Total Polar Surface Area; [h] iLogP, octanol/water partition coefficient; [i] HIA%, human gastrointestinal absorption; [j] BBB permeant, Blood-Brain Barrier penetration; [k] Pgp substrate, Permeability glycoprotein; [l] F, Abbott oral bioavailability score; [m] PAINS, Pan-Assay Interference Compounds.

Information Classification: General
